# Mycorrhizal fungal associations of the fringed orchids (*Platanthera*) in the US, inter- and intra-species variation

**DOI:** 10.1007/s10531-025-03233-4

**Published:** 2026-01-23

**Authors:** Melissa K. McCormick, Ida Hartvig, Simone A. Evans, Julianne McGuinness, Stephanie Lyon, Zöe Knapp, Jason Ligon, Lauren Eserman, Emily Coffey, Dennis F. Whigham

**Affiliations:** 1 North American Orchid Conservation Center, Smithsonian Environmental Research Center, Smithsonian Institution, 647 Contees Wharf Road, Edgewater, MD 21037 USA; 2 Center for Evolutionary Hologenomics, Globe Institute, University of Copenhagen, Øster Farimagsgade 5, 1353 København C, Denmark; 3 Department of Genetics, Stanford University, Stanford, CA 94305 USA; 4 Department of Biology, University of Wisconsin, Stevens Point, 2100 Main Street, Stevens Point, WI, 54481-3897 USA; 5 Australian Capital Territory, Australian National Botanic Gardens, GPO Box 1777, Canberra, 2601 Australia; 6 Atlanta Botanical Garden, 1345 Piedmont Avenue, Atlanta, GA 30309 USA

**Keywords:** Orchid mycorrhizal fungi, Orchid conservation, Mycorrhizal specificity, Hybridization

## Abstract

Understanding which orchid mycorrhizal fungi (OMF) are needed to support orchid growth is critical for their conservation. We used DNA sequencing and network analysis of OMF collections from the North American Orchid Conservation Center, along with published sequences, to compare the OMF associated with 28 species and 4 natural hybrids in the genus *Platanthera*. We found that relatedness of orchid species was generally a poor predictor of OMF associates. However, species in sections *Fimbriata* and *Blephariglottis* that co-occurred and formed natural hybrids associated with multiple fungi that were completely shared among co-occurring species. Differences in mycorrhizal associates within species mapped onto orchid genetic differences that distinguished geographic regions (mountains vs. coast vs. inland, not strictly geographic distance). Orchids that were specialized or moderately specialized in their OMF associations required relatively few samples to describe the bulk of their associated OMF, while generalized orchids required considerably more samples to describe their associated OMF. These results provide insight into species differences in mycorrhizal associations and can be used to guide management actions, as well as providing a foundation for future research.

**Supplementary Information:**

The online version contains supplementary material available at 10.1007/s10531-025-03233-4.

## Introduction

Most plants depend on symbionts, especially mycorrhizal fungi and pollinators, to complete their life cycles (Sexton et al. 2009), which means that effective plant conservation also needs to involve conservation of required symbionts (Carthey et al. 2020; Limborg et al. 2024; Walling et al. 2025). For orchids, a critical part of their ecology is an obligate dependence on mycorrhizal fungi. Indeed, the complex interactions orchids maintain with often specific mycorrhizal fungi and pollinators are thought to drive a high risk of extinction (Dixon et al. 2007; Orejuela-Gartner 2012) and may contribute to them being among the first plants to decline when habitats are degraded (Reiter et al. 2016).

Associations with mycorrhizal fungi mediate growth, competitive interactions, and pathogen protection for the vast majority of terrestrial plants, but the ability of fungi to drive plant dynamics is perhaps most likely to be seen in orchid mycorrhizal associations (Swarts and Dixon 2017). Mycorrhizal associations are essential for orchid recruitment from seed (Rasmussen 1995), and they remain an important component of orchid nutrition and ability to tolerate stressful conditions throughout their lives (McCormick et al. 2022). Additionally, orchid emergence and flowering have been found to reflect the abundance of their mycorrhizal fungi (Rock-Blake et al. 2017; McCormick et al. 2018; Whigham et al. 2021). Associating with different mycorrhizal fungi may also contribute to keeping species distinct or promoting speciation (e.g., Jacquemyn et al. 2014). In contrast, overlapping mycorrhizal associations can allow species to co-occur, which may promote hybridization, with implications for speciation, adaptation to changing conditions, or possible merging of species (Wong et al. 2022). All these possibilities are important to consider for conservation. Consequently, successful orchid conservation also requires conservation of mycorrhizal fungi.

Orchid mycorrhizae have been studied for more than a century (Rasmussen 2002) and the orchid mycorrhizal association is generally considered to be obligate for the orchids, with the fungi maintaining independent distributions. Orchids form mycorrhizal associations with phylogenetically and ecologically diverse fungi, but the most commonly orchid-associated fungal genera are *Tulasnella*, *Ceratobasidium*, and *Serendipita* (Dearnaley et al. 2012). All orchids at the protocorm stage are initially mycoheterotrophic (Leake 1994; Rasmussen 2002), relying entirely on orchid mycorrhizal fungi (OMF). At the seedling stage (Whigham et al. 2008) and beyond, orchids interact with OMF to varying degrees (Girlanda et al. 2011; Selosse and Martos 2014; Stöckel et al. 2014).

Despite the importance of symbiotic associations, especially mycorrhizae, conservation efforts rarely consider symbiotic partnerships. For example, in a survey of 130 management plans in the U.S., Markovchick et al. (2023) found that 92% ignored mycorrhizae. It is often assumed that target species will acquire needed symbiotic partners when they are introduced (Rawat et al. 2022), but for species that depend on specific symbiotic partnerships, such as orchids (Markovchick et al. 2023), this assumption may result in unsuccessful restoration efforts. Research has demonstrated that restoring native mycorrhizal fungal communities in conjunction with native plant communities can increase planting survival and establishment, native plant biomass, plant species richness, and resulting community similarity to reference ecosystems, resulting in restoration success improvements of 30% or more (Rua et al. 2016; Koziol and Bever 2017; Neuenkamp et al. 2019; Policelli et al. 2020).

Because orchids are obligately dependent on their mycorrhizal fungi in natural environments, the distribution of fungi has a considerable role in determining where orchids occur and whether conservation efforts are successful (McCormick et al. 2016, Hartvig et al. 2024). Despite their unquestioned importance for orchid ecology, OMF are still only occasionally explicitly considered in orchid conservation efforts. Reiter et al. (2016), in a review of Australian orchid translocations, found that only 17 studies germinated plants symbiotically and only five of 74 studies verified the presence of appropriate mycorrhizal fungi at recipient sites. However, they also found that ensuring the presence of appropriate mycorrhizal fungi and pollinators was a key determinant of reintroduction success. Fourteen of 17 studies that symbiotically germinated plants had survival of > 50%. Planting symbiotically grown orchid plants is generally thought to have higher restoration success than adding seeds, but that comes with a significant investment of time and expense. It would be far easier if germination of added seeds could be improved, and OMF may have an even more critical role here. McCormick et al. (2012) found that OMF addition, with organic amendments, supported higher seed germination in three species of forest orchids, and Těšitelová et al. (2022) found that adding OMF inoculum increased seed germination in orchid restoration efforts. The few studies that have examined OMF distribution over large scales have suggested that most OMF are geographically widespread, and their availability is not thought to be a factor in determining orchid range size (Swarts et al. 2010; Phillips et al. 2011), but the local presence and abundance of appropriate OMF are thought to be major determinants of whether an orchid can grow in a given site (e.g., Rock-Blake et al. 2017; McCormick et al. 2018; Whigham et al. 2021). However, there is a notable disconnect between the science to understand mycorrhizal fungus contributions to plants and ecosystems and conservation planning and implementation (Markovchick et al. 2023).

The first step to incorporate OMF in orchid conservation is knowing which fungi associate with which orchids. But this step represents one of the major impediments to incorporation of mycorrhizal fungi into orchid conservation, along with the isolation, culture, and sustainable production of the mycorrhizal fungi. It can be prohibitively time-consuming and technically challenging for conservation organizations to conduct a thorough assessment of the fungi associated with a target orchid, particularly when it is unclear how much effort such an assessment would take.

To facilitate effective integration of OMF in orchid conservation actions, we sought to determine whether we can predict pairings between OMF and individual orchid taxa, based on which OMF associated with closely-related species, whether an OMF from another site will associate with an orchid in a new location, and how extensive sampling needs to be to assess the diversity of fungi that can associate with an orchid taxon.

We used OMF associated with orchids in the genus *Platanthera* to determine what is needed to identify the OMF associated with 21 different taxa. Specifically, we predicted that (1) OMF identity will reflect the relatedness of orchid taxa, such that the OMF associated with one species will be predicted by the OMF associated with closely related taxa, and (2) OMF associated with an orchid taxon at one site will be more similar to those at geographically close sites, compared to those at sites farther away. We further use sampling of the OMF associated with multiple species of *Platanthera* to predict (3) the amount of sampling of individuals within and among sites that is required to describe the OMF associated with individual orchid taxa.

## Methods

### Study system

The terrestrial orchid genus *Platanthera* (Rich.) (Richard 1817) is an ideal system for studying how the identity and specificity of associations with mycorrhizal fungi are related to orchid phylogenetic relationships. With approximately 45 species in North America (https://goorchids.northamericanorchidcenter.org/genus/platanthera/), and 100–150 species across the temperate northern hemisphere (Efimov 2016), the genus exemplifies the extensive radiation that has contributed to orchids being one of the most species rich vascular plant families. The genus maintains considerable diversity, but also includes many species with overlapping spatial distributions, some of which form natural hybrids (Wallace 2006; Bateman et al. 2012; Runemark et al. 2019) that include products of both polyploid and homoploid hybridization (Wettewa et al. 2020).

Hapeman & Inoue (1997) divided *Platanthera* into 5 well-supported subgenera, and Efimov (2016) further divided two of the subgenera (subgen. *Platanthera* and *Blephariglottis*) into 9 and 2 sections, respectively. Within two of these sections (*P.* subgen. *Blephariglottis* section *Blephariglottis* and *P.* subgen. *Platanthera* section *Fimbriata*), there are groups of species that form natural hybrids, as well as recognized species that are purported to be of hybrid origin (Evans et al. 2023, Hartvig et al. 2022). Previous explorations of mycorrhizal diversity within these two sections found that the species within *P*. (subgen. *Blephariglottis* section *Blephariglottis*) associated primarily with OMF in the genus *Tulasnella* (Zettler and McInnis 1994; Zettler and Hofer 1998; Zale et al. 2022), while two species within *P*. (subgen. *Platanthera*) Section *Fimbriata* associated primarily with fungi in the genus *Ceratobasidium* (Zettler et al. 2005). While we conducted broad sampling throughout the genus, we focused on these two groups to better understand similarities among closely-related species.

### Sample collection

From 1998 to 2024, sampling as independent researchers and as part of efforts coordinated by the North American Orchid Conservation Center (NAOCC), we collected 558 root samples from 28 *Platanthera* species and 4 hybrids. All samples were collected with all appropriate permissions from federal, state and/or local authorities, as well as from private landowners, where relevant, and are retained in NAOCC records. For each root, we attempted to isolate OMF into pure cultures, by isolation and plating of fungal pelotons, as per McCormick et al. (2004). In each case, when isolation was attempted, we retained 0.25–1.25 cm^3^ of remnant root tissue containing OMF pelotons and froze it at −20 °C in water or 2x CTAB for later analysis. A complete list of taxa and localities included in the study, along with which had sequences obtained from cultures, Sanger sequencing from root remnants, and metabarcoding, can be found in Supporting information (Table S1), as can the number of samples of each species analyzed by each sequencing method (Table S2).

### Sequencing cultured fungi

 When pelotons grew, forming pure colonies of presumed OMF, we transferred a subculture of each fungus into liquid E-medium (Caldwell et al. 2000) for 2–4 weeks to provide sufficient pure tissue for DNA extraction. DNA was extracted from ~ 0.25 cm^3^ of fungal tissue using CTAB extraction (McCormick et al. 2004), Qiagen BioSprint96 kits (Qiagen, Inc., Valencia, California), Qiagen DNEasy Plant kits (Qiagen, Inc.), or Nucleospin kits (Macherey-Nagel Inc., Allentown, PA, USA), following the supplied protocols. We identified each cultured fungus using Sanger sequencing of the internal transcribed spacer of the nuclear ribosomal repeat (hereafter ITS), using primers ITS5/ITS4, followed by a BLAST search in GenBank, as per McCormick et al. (2004).

### Sequencing OMF in roots (Sanger method)

For all cases where isolation was unsuccessful, cultures became irrevocably contaminated, or if only non-OMF were cultured, we extracted DNA from the frozen root remnants, using one of the same DNA extraction methods as for cultured fungi. Earlier (i.e., 1998 to approximately 2019) attempts to identify OMF from root remnants using Sanger Sequencing included PCR with primers that were specific to *Tulasnella* (ITS5/ITS4Tul, White et al. 1990; Taylor and McCormick 2008) and specific to *Ceratobasidium* (CeTh1/CeTh4, Porras-Alfaro and Bayman 2007), as well as with general fungal primers ITS1F/ITS4 (White et al. 1990; Gardes and Bruns 1993), plus a nested PCR with ITS5/ITS4, followed by amplification with ITS3Seb/ITS4 (Ray et al. 2015; Gardes and Bruns 1993). The nested Sebacinales reaction was necessary because general fungal primers often preferentially amplify non-mycorrhizal fungi instead of Sebacinales or amplify multiple fungi, resulting in mixed sequences for Sanger sequencing. Existing Sebacinales primers do not amplify strongly, while ITS5/ITS4 amplifies plant DNA, in addition to the target fungal DNA. Amplification was performed with PCR conditions as in each source publication, using a Bio-Rad C1000 Touch Thermal Cycler (Bio-Rad Laboratories, Inc., Hercules, CA). Sanger sequencing was carried out as per Downing et al. (2020).

### Sequencing OMF in roots (metabarcoding)

After 2019, identification of OMF in root remnants, including some older root remnants from which we were unable to obtain clean OMF sequences using Sanger Sequencing, and all newer root remnants, utilized metabarcoding analysis on an Illumina MiSeq (Illumina, Inc. San Diego, CA, USA). For these samples, DNA that was extracted using DNEasy Plant kits (Qiagen, Inc.) or Nucleospin kits (Macherey-Nagel Inc., Allentown, PA, USA) was subjected to PCR using Apex Taq RED Mix (Apex Bioresearch Products) in 15 µl reactions (1 µl template DNA, 7.5 µl RED 2X master mix, 0.045 µl each primer (100 µM), 0.15 µl BSA, and 6.26 µl sterile DI water), with two different primer sets: ITS5/ITS4Tul and ITS1F/ITS4. All primers included a Nextera tail to allow later joining to barcode indexes. Following PCR, 1 µl of each resulting product was visualized on a 1% agarose gel after electrophoresis in the presence of GelRed. All reactions included a template-free negative control to verify lack of contamination.

For each sample, the ITS5/ITS4Tul product, which we used to ensure representation of *Tulasnella* DNA, which is poorly represented in metabarcoding analyses that use only general fungal primers (Rammitsu et al. 2021), was combined 1:3 by volume with product from ITS1F/ITS4 to produce a single fungal PCR product. We have found that increasing ITS5/ITS4Tul product above 1:3 does not increase the diversity of *Tulasnella* that are detected. While increasing the proportion of the general fungal PCR product does increase the diversity of fungi detected, it has little effect on the diversity of OMF that we detected. Each combined sample was then cleaned using ExoSAP-IT Express (Applied Biosystems). A unique combination of Nextera i5 and i7 barcode indexes was attached to each sample using PCR (95 °C for 5 min, followed by 8 cycles of 95 °C for 30 s, 55 °C for 30 s, and 72 °C for 30 s) in reactions containing 2 µl template DNA, 12.5 µl RED Mix, 0.75 µl i7 index (10 µM), 0.75 µl i5 index (10 µM), 0.25 µl BSA, and 8.75 µl sterile DI water, totaling 25 µl.

Each indexed PCR product was cleaned using ExoSap-IT™ (Applied Biosystems) and products were pooled together in equimolar concentration. A final cleanup was conducted on the resulting library using KAPA Pure Beads (Roche), with a 0.8X bead to library ratio. The library was analyzed on an Agilent TapeStation 4200 (Agilent, Santa Clara, CA, USA) to check for the expected DNA fragment size range and sequenced using paired end reads on an Illumina MiSeq by the Laboratories of Analytical Biology at the Smithsonian National Museum of Natural History, using an Illumina MiSeq Reagent Kit v2 (300 bp, 500 cycles).

### Identifying OMF

Sanger sequences were manually checked for quality and trimmed. Forward and reverse sequences were combined into single contigs, and discrepancies resolved manually in Sequencher v.5.4.6 (Gene Codes Corp., Ann Arbor, MI). Because of difficulty aligning ITS sequences across distantly related taxa, we first used BLAST in the NCBI Genbank database to identify sequences to genus and then further analyzed sequences separately by genus (*Ceratobasidium*, *Serendipita*, and *Tulasnella*). Note that all Sebacinales were considered, but our sequences all fell within *Serendipita* in our BLAST searches, so we have only listed that genus here.

Root sequences from metabarcoding molecular analysis were processed with the QIIME2 2023.2 pipeline (Bolyen et al. 2019). Briefly, sequences were demultiplexed using Demux, primers were removed using Cutadapt (Martin 2011), and sequences were quality checked and trimmed using DADA2 (Callahan et al. 2016). Although reads were sequenced as paired end reads, the forward and reverse reads were not paired because the sequenced region was ~ 600–800 bp and reads were too short (240 bp) to overlap. Instead, forward and reverse reads were analyzed separately for community composition and to ensure that OMF taxa representing at least 10% of the reads were similarly represented in both forward and reverse reads. The community composition obtained from forward and reverse primers was qualitatively and quantitatively similar. As a result, we used forward reads for *Tulasnella* analyses, because more forward reads passed sequence quality checks. However, we used reverse reads for Sebacinales sequences, to better align with the ITS2 Sanger sequences produced by the Sebacinales-specific primers. For *Ceratobasidium*, neither forward, nor reverse sequences from ITS1F/ITS4 primers aligned well with the sequences generated by *Ceratobasidium*-specific (CeTh1/CeTh4) primers. However, forward sequences overlapped with the Sanger sequences by ~ 103 bp, so we used this short region, in alignment with reference sequences, to compare to full-length sequences. Taxon identification generally focused on the full-length sequences to which the shorter sequences matched, as they were more taxonomically informative.

Because we were interested in the most abundant OMF, we considered only OTUs that constituted at least 10% of the metabarcoding reads for each root. This was based on the assumption that the most important OMF will also be among the most abundant in the roots and reflects the finding of Mennicken et al. (2024) that the OMF that were the most abundant and mostly likely to be cultured in orchid roots were also represented by the most metabarcoding reads. This criterion generally produced 1–2, occasionally 3, OMF OTUs per root, which was similar to what we obtained with Sanger sequencing using multiple specific primers. This also allowed us to minimize potential differences between DNA extraction methods.

A pre-trained dynamic UNITE classifier (unite_ver10_dynamic_04.04.2024-Q2-2024.2.qza; Colin Brislawn; https://github.com/colinbrislawn/unite-train/releases) was used to classify the sequences into OTUs of 97–99% similarity. We created feature tables with the number of sequences belonging to each potential OMF OTU (i.e., those in *Tulasnella*, *Ceratobasidium*, or *Serendipita*) that were found in each sample. All OMF sequences that were represented in > 10% of the reads for a sample were imported into Geneious Prime 2020.0.4 (BioMatters Ltd.) to determine their similarity to Sanger sequences from orchid roots.

### Assembly of published sequences

In addition to the sequences that we generated, we also obtained additional ITS sequences from Genbank (accessed Dec. 12, 2024) that had come from OMF in North American *Platanthera* species. These were identified by searching publication databases for “(*Platanthera* OR *Piperia*) AND (Mycorrhiza* OR Fung*)”, which we then screened to include only North American *Platanthera* species and those with OMF that were identified using ITS sequences. We identified all Genbank accession numbers listed in these papers and obtained the sequences from NCBI. This allowed us to clearly identify all sequences of OMF from published papers, since sequences deposited into Genbank do not always include host information. Additionally, to identify sequences that had been entered into GenBank, but not associated with a published paper, we searched the Nucleotide database in NCBI for “(*Platanthera* OR *Piperia*) AND (*Tulasnella* OR *Epulorhiza* OR *Ceratobasidium* OR *Ceratorhiza* OR *Serendipita* OR *Sebacina*)” and screening for ITS sequences. Together, these searches yielded sequences from four additional orchid species for which we had few (*P. praeclara*, *P. chapmanii*; Kaur et al. 2017, 2019) or no (*P. cooperi*, *P. yadonii*; Kaur et al. 2021; Pandey et al. 2013) OMF sequences. To these, we added 16 *P. leucophaea* sequences that had been published (Thixton et al. 2020), but not previously entered into GenBank (obtained from H.L. Thixton).

### Similarity of OMF among Orchid species

Keeping fungal genera in separate files, to avoid challenges in determining homologous regions in the highly variable ITS1 and ITS2 regions, we combined Sanger and metabarcoding reads. To provide a reference to named OMF species, we added Genbank sequences from 31 named *Tulasnella* fungi, 23 sequences from named *Ceratobasidium* fungi, and 11 sequences from named *Serendipita* fungi to use as references for our phylogenetic trees. We aligned the full length of all OTUs using Clustal Omega, implemented in Geneious and checked alignments manually. We then estimated phylogenetic trees for each genus using FastTree 2.1.11 (fastest setting, GTR model, rate categories of sites = 20) in Geneious. We also obtained patristic genetic distances between all pairs of OMF sequences to use for our mantel analysis of the relationship between geographic distances between populations and OMF genetic similarity.

To assess the extent to which OMF were shared among orchids, we used Geneious denovo alignment (maximum 10% gaps, min 70% overlap, word length 24, minimum overlap identity = 97%, index word length 14, reanalyze threshold 16, maximum ambiguity 10) to group the sequences into OTUs, with 97% similarity as the cutoff for inclusion. This allowed us to group similar sequences from Sanger sequencing and from metabarcoding, despite the differences in sequence length. Ninety-five and 99% similarity were also tested, but results were similar, so only OTUs clustered at 97% are presented here. These OTUs were then used for the network analysis that we used to estimate specificity and determine the extent to which OMF were shared among orchids.

To conduct network and modularity analyses, we used Bipartite (Dormann et al. 2008), implemented in R version 4.4.2 (R Development Core Team 2023) in RStudio (2024.12.0, build 467, 2009–2024 Posit Software, PBC). We used network-level specialization (*H*_*2*_*’*) to describe the extent to which orchids associated with distinct groups of OMF (Blüthgen et al. 2006). We used modularity to represent the degree of clustering in the network into distinct communities (Guimerà et al. 2004; Guimerà and Amaral 2004). Both analyses were conducted using metrics that weighted taxa by the number of samples in which they occurred, not by the number of reads. We restricted our network analysis to the 19 species and 2 hybrids for which we had 6 or more samples, to avoid having results skewed by a small number of samples with undue influence on species-level interpretations. Using this dataset, we calculated network-level specificity (*H*_*2*_*’*) using the H2fun function (Blüthgen et al. 2006) and modularity (Q) using ComputeModules (QuaBiMo, Dormann and Strauß 2014) in *bipartite* package (Dormann et al. 2008) in R version 4.4.2 (R Development Core Team 2023). The significance of each statistic was computed by comparison to statistics calculated on 1000 null model permutations for each analyzed network, which were generated using the swap.web algorithm. Swap.web constrains network marginal totals and connectance, making it a conservative null model. We used the clusters identified by the modularity analysis to determine which orchids shared OMF.

### Geographic and genetic similarity

To determine whether orchids at nearby sites had more similar OMF communities than more distant sites, we calculated Mantel’s r for OMF genetic distance as a function of geographic distance using geodist (Padgham and Sumner 2024) in R. To avoid obscuring patterns within a fungal genus with the large genetic distances among OMF genera, as well as problems with comparing the highly variable ITS sequences in *Tulasnella* with the less variable sequences within *Ceratobasidium*, we conducted this analysis separately for *Tulasnella* and *Ceratobasidium* within each orchid species.

For the species that had a significant correlation between geographic and genetic distance for either genus, we also conducted a modularity analysis, as described above, within each of the orchid species and groups, to determine whether there was significant within-species modularity that corresponded to orchid genetic groups found by Hartvig et al. (2026). These groups were *P. blephariglottis*/*P. ciliaris*/*P. x bicolor*, and *P. grandiflora*/*P. psycodes*/*P. shriveri*.

### Required sampling intensity

To determine the amount of sampling within and among sites that was needed to describe the breadth of OMF associated with each species, we first computed the species specificity index (*d’*) using dfun (Dormann 2011) in *bipartite* package (Dormann et al. 2008) in R version 4.4.2 (R Development Core Team 2023) for each orchid species to distinguish more general and more specific orchids. We considered species with *d’* *≥* 0.70 to be specialized, 0.5 < *d’* < 0.7 to be moderately specialized, with the remainder being generalized. We then calculated the number of Effective Partners using ComputeModules to estimate the number of OMF OTUs associated with each orchid. Next, we generated species accumulation curves using SpecAccum in VEGAN (Oksanen et al. 2025) in R to determine whether species estimates from our sampling adequately described the total diversity associated with each species. We used these indexes to place the study orchid species into three categories: Generalist, Moderate Specialist, and Specialist. While we recognize that specificity is a continuous variable, we feel that categorization makes applying the findings of this work to conservation easier. Together, these analyses indicate how much sampling is needed to determine OMF identity, depending on how specific the OMF association of a target orchid is.

## Results

We obtained 1235 OMF sequences, consisting of 707 *Tulasnella* sequences, 505 *Ceratobasidium* sequences, and 23 *Serendipita* sequences from 28 *Platanthera* species and 4 hybrids. These sequences were grouped into 105 *Tulasnella* OTUs, 29 *Ceratobasidium* OTUs, and 4 *Serendipita* OTUs, of which 64 *Tulasnella*, 6 *Ceratobasidium*, and 2 *Serendipita* were only identified from a single root. These sequences were obtained from 509 orchid roots, plus 624 previously published sequences from 36 individuals of *P. chapmanii* (Kaur et al. 2017), 176 of *P. cooperi* (Kaur et al. 2021), 148 of *P. praeclara* (Kaur et al. 2019), and 42 of *P. yadonii* (Pandey et al. 2013) (Fig. S1).

Although there were costs and benefits to each method, the different sequencing methods were roughly equivalent in the numbers of distinct OMF taxa we identified in orchid roots and the distribution of OMF between *Ceratobasidium* and *Tulasnella* (Table S2). Sanger sequencing produced a mean of 1.25 OTUs per root, culturing produced 1.17 OTUs per root, and metabarcoding with a 10% cutoff produced 1.31 OTUs per root. Orchid species in different sections had different “preferences” for *Tulasnella* versus *Ceratobasidium* OMF (Fig. S2). All three sequencing methods also revealed fungi that were presumed endophytes or pathogens that did not belong to the standard OMF taxa that were not investigated in this paper.

### Similarity of OMF among Orchid species

**Fig. 1:**
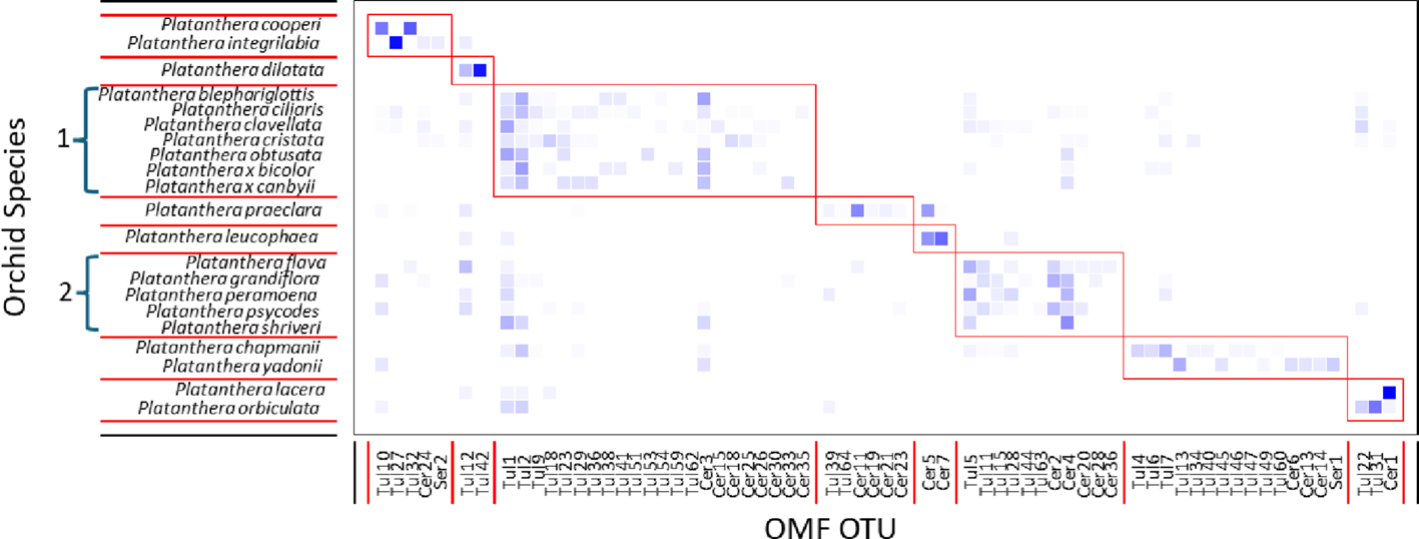
A typical modularity plot, generated by ComputeModules in Bipartite. The darkness of blue squares indicates the proportion of orchid root samples each OTU was associated with. Orchid species are arrayed on the left side of the plot, with the OMF OTUs arrayed across the bottom of the plot. The two consistent modules of closely related species are indicated by brackets and numbered 1 and 2. The composition of other modules varied among runs

Most *Platanthera* species associated with distinct communities of OMF. Networks of orchid-OMF interactions were highly significantly modular (Q = 0.57, *P* = 2.0 × 10^− 34^; Fig. 1) and specialized (*H*_*2*_*’*=0.54, *P* = 4.85 × 10^− 45^), indicating that groups of orchids associated with distinct groups of OMF and that specialists largely associated with a subset of the species that more general species associated with.

Orchids that co-occurred (see Table S1 for co-occurrence information), especially those that formed natural hybrids and associated with overlapping fungal communities, were grouped into the same modules (Fig. 1). This was particularly apparent within section *Blephariglottis*, where most species associated with OMF that clustered into a single module (module 1 in Fig. 1), with *P. cristata*, being somewhat distinct from the rest of the section, and *P. chapmanii*, which potentially originated as a hybrid between *P. cristata* and *P. ciliaris* (but see Hartvig et al. 2026), shifting among modules between runs (see Fig. S3). This module was robust and was retained across multiple runs (Fig. S3), as well as when the analysis was conducted with only samples from single-species populations, excluding populations with extensive apparent hybridization (data not shown). However, the overlap of OMF did not apply to the entire section. Within section *Blephariglottis*, *P. integrilabia* associated with OMF that were distantly related to the *Tulasnella* taxa that commonly associated with the rest of the orchids and it associated with a very narrow range of OMF. Notably, *P. integrilabia* is also less closely related to the other orchids in the section (Hartvig et al., 2026). Similarly, the purple fringed orchids of section *Fimbriata* all associated with overlapping OMF communities and were clustered into a single module (module 2 in Fig. 1). Similar to section *Blephariglottis*, section *Fimbriata* contained one orchid species, *P. lacera*, that associated with a very narrow range of OMF OTUs that were distinct from the fungi in association with the other species the section. For section *Fimbriata*, *P. lacera* is also less closely related to the taxa that consistently shared fungi (*P. grandiflora*/*P. psycodes*/*P. shriveri*, Figure S3, Hartvig et al., 2026).

In addition to indicating overlapping OMF associations among very closely related species, the modularity analysis also revealed some orchids that associated with a single dominant OMF OTU or with a narrow range of OTUs that were often distinct from those associated with other orchids. These visible distinctions roughly mirrored the species specificity index (*d’*) and the effective species number. Orchids with a single particularly common OTU (indicated by darker shades of blue in Fig. 1) also had a very high *d’* and low effective species number (Table 1).

**Table 1:**
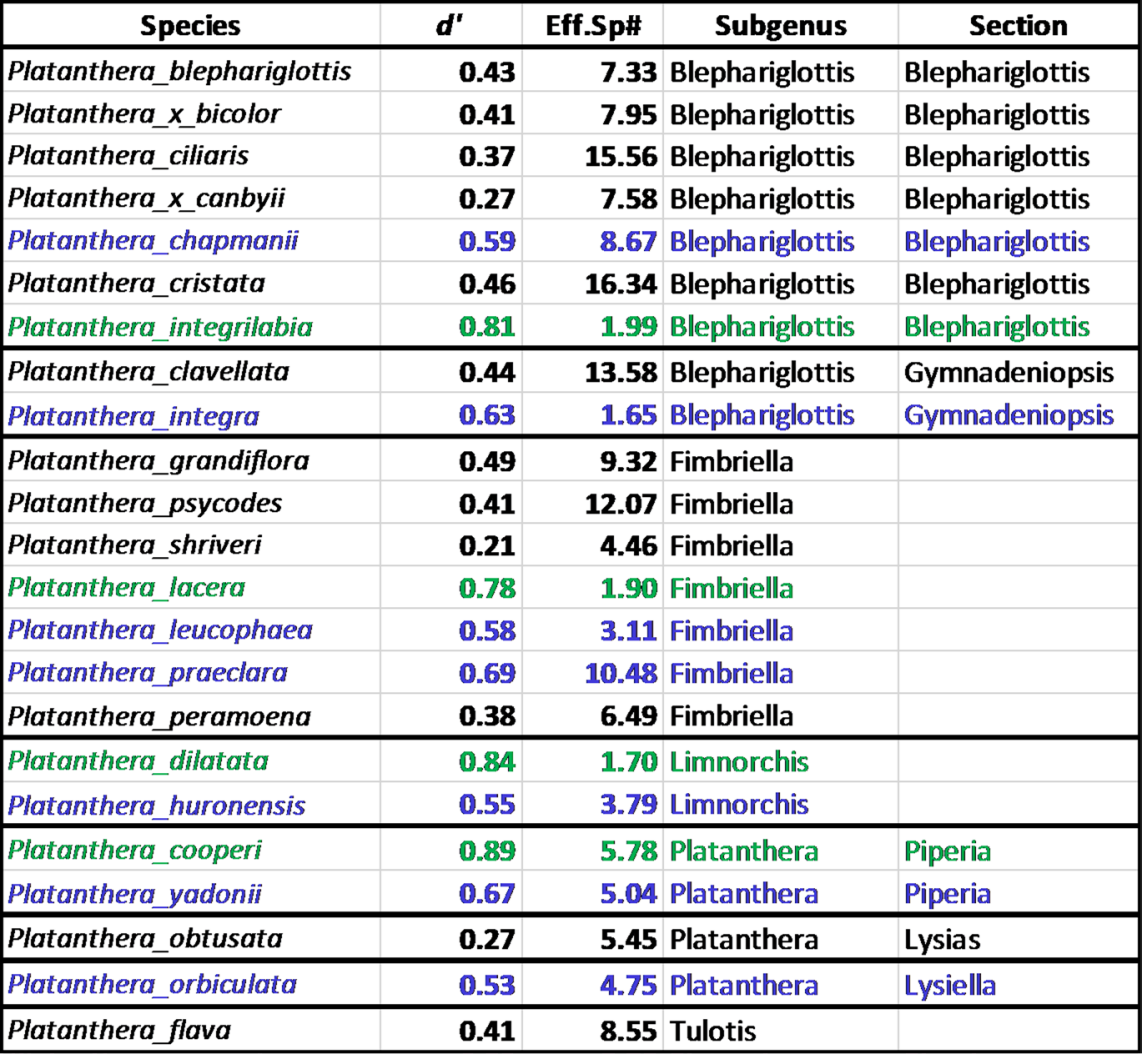
Two indications of specificity in OMF associations indicate the species specificity index (d’) and number of effective species (Eff.Sp#). Species are color coded as specialists (d’ > 0.7, green text) moderate specialists (0.5 < d’ < 0.7, blue text) or generalists (d’ < 0.5, black text), based on d’ values

Darker borders in the table separate orchids in different sections within *Platanthera*

**Fig. 2:**
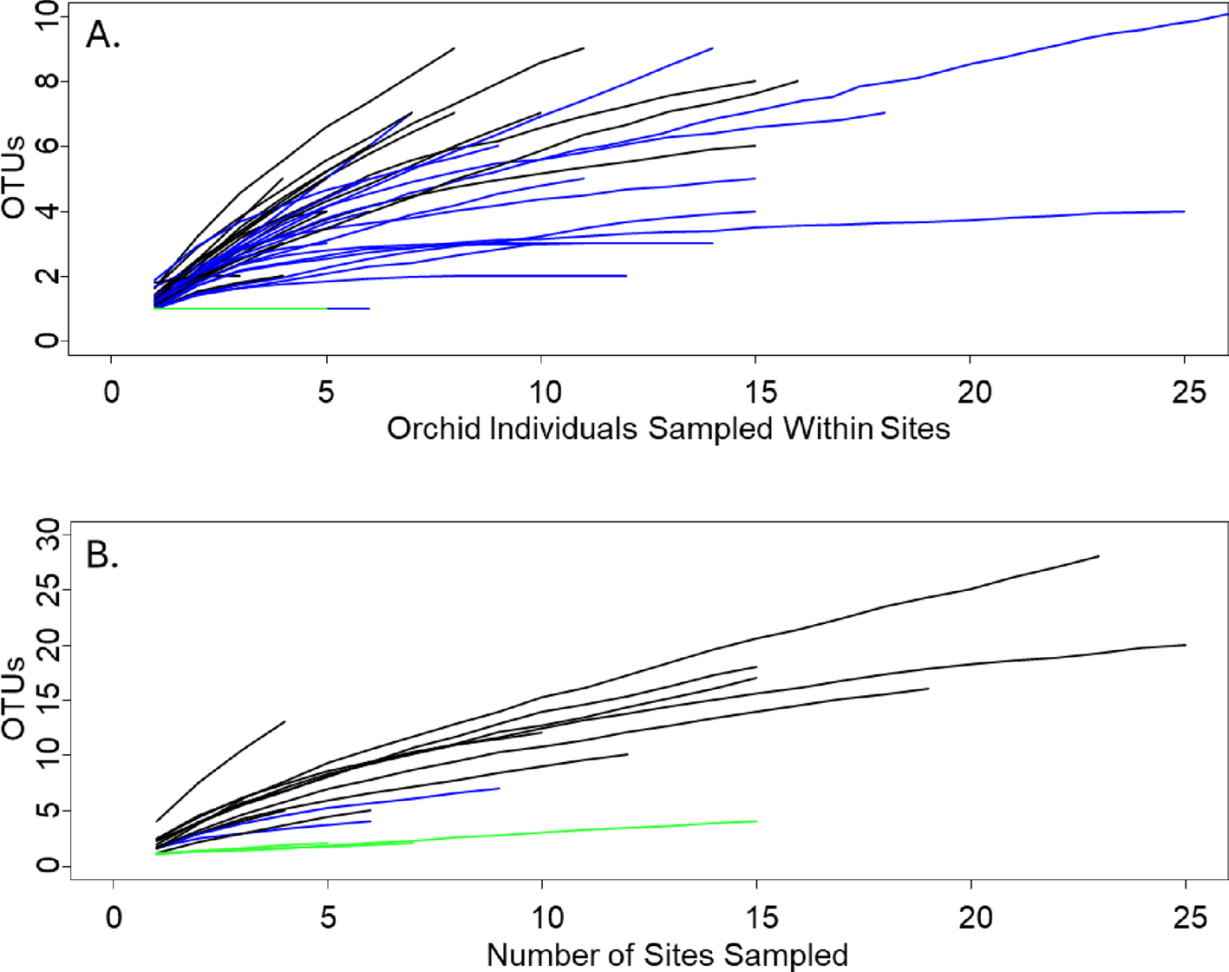
Species accumulation curves for (**A**) orchid individuals sampled within sites (each line is one site), and (**B**) number of sites sampled (each line is one species, so two species sampled at the same site contribute to different curves). Both within and among sites, most specialized (green lines) and moderately specialized (blue lines) associations approached an asymptote within 10 or fewer samples, while generalized species (black lines) continued to increase

### Geographic and genetic similarity

We found limited evidence for geographic patterns (See Table S1 for geographic location information) in OMF associations, suggesting that nearby populations associated with OMF communities that were no more similar than populations that were farther apart. Most species had non-significant Mantel statistics (Table 2) and associated with similar OMF throughout their ranges, but one species and one hybrid, *P. cristata* and *P. x bicolor* did have significant geographic by genetic distance relationships in *Tulasnella* and two species, *P. ciliaris* and *P. clavellata*, had significant Mantel statistics for *Ceratobasidium*. For these, we conducted modularity analyses within each species or species group, with divisions based on genetic groupings from Hartvig et al. (2026) to determine whether significant geographic patterns of OMF association aligned with patterns of orchid genetic groups.

Modularity analyses revealed significant clustering of OMF communities aligned with genetic groupings that divided coastal, mountain, and inland populations of *P. cristata* (Q = 0.469, *P* < 0.001) and the group of *P. blephariglottis*/*P. ciliaris*/*P. x bicolor* (Q = 0.58, *P* = 8.8 × 10^− 24^). Dividing these species into those growing in single species populations versus those co-occurring and hybridizing with other species produced non-significant modularity values (both Q < 0.40 and *P* > 0.1). In contrast, when analyzed alone, *P. ciliaris* showed less significant variation in genetic composition (Hartvig et al. 2026), and its OMF groups did not form modules corresponding to genetic groupings. This suggests that the identity of OMF associates may reflect genetic distinctions within and among species, but that geographically closer sites did not otherwise have more similar OMF than more distant sites.

**Table 2:** Results of mantel analyses for geographic autocorrelation in genetic similarity of OMF associated with Orchid species

Species	Tulasnella		Ceratobasidium	
	Mantel’s r	p-value	Mantel’s r	p-value
*P. blephariglottis*	0.065	0.219	−0.014	0.454
*P. ciliaris*	0.040	0.189	0.214	**0.019**
*P. x bicolor*	0.299	**0.007**	−0.038	0.507
*P. bleph*,* cil*,* xbic*	0.050	0.136	0.141	**0.014**
*P. cristata*	0.270	**0.003**	0.051	0.334
*P. clavellata*	−0.647	0.851	0.114	0.053
*P. grandiflora*	−0.154	0.964	−0.137	0.808
*P. psycodes*	−0.004	0.532	−0.059	0.816
*P. grand*,* psyc*,* shri*	−0.035	0.694	−0.009	0.527

### Required sampling intensity

With the exception of the two groups of closely related orchids that co-occurred and hybridized, we did not find evidence that more closely related orchids could be used to predict the mycorrhizal fungi needed by a different orchid. Similarly, we found that within a species, OMF at nearby sites were not any more similar than those at more distant sites.

Species accumulation curves calculated from sampling individuals within sites (Fig. 2A) and from sampling multiple sites (Fig. 2B) for each orchid species indicated that OMF diversity within sites often reached an asymptote within approximately 5 samples for specialist orchids and within 5–10 samples for moderate specialists. OMF diversity associated with generalized species had not yet reached an asymptote with 20 samples in a site. Across sites, the pattern was similar to findings within sites, but with many more taxa identified when multiple sites were sampled than when multiple individuals within a site were sampled.

## Discussion

We found that the most closely related *Platanthera* species often associated with similar groups of OMF. We also found that the overlap of OMF communities was most common among *Platanthera* species that co-occurred and often formed natural hybrids. Outside of these closely related and co-occurring orchids, there was often little overlap in OMF associations between more distantly related *Platanthera* species. OMF also differed among sites for all but the most specific *Platanthera* species. As a result, OMF associated with *Platanthera* species at different sites could rarely be used to identify OMF of a species of particular interest. We also found that the number of samples needed to identify which OMF associated with an orchid depended on how specialized an orchid was on particular OMF. This suggests that it will be necessary to sample each orchid species to determine OMF associates, but for some species, this might be achievable with relatively few samples.

We used diverse DNA extraction and sequencing methods to assess the OMF that associated with each orchid. Certainly, the sequencing methods produce different numbers of fungi in orchid roots, with metabarcoding regularly identifying many non-OMF fungi. By limiting our assessment to known OMF taxa that represented 10+% of the reads in a sample, we obtained similar numbers of OMF that likely represented the dominant taxa in the orchid roots. It is possible that we missed taxa that were less common in the root fragments we assessed or that belonged to non-standard OMF genera. However, in examining the fungi that were sequenced by metabarcoding, there were no taxa other than *Tulasnella*, *Ceratobasidium*, or *Serendipita* that were shared among more than two samples, suggesting that, for this orchid genus, restricting the OMF genera may be a reasonable constraint to place on sequence interpretation. Where we used multiple methods to identify the OMF in a single root (Table S2), we always identified the same OMF, suggesting that, with the limitations we used, the methods are robust.

### Similarity of OMF among Orchid species

If mutualisms are important drivers of speciation, then recently diverged species should tend to differ in their mutualistic partners or the nature of their interaction with their partners (e.g., Waterman et al. 2011). Although co-diversification of orchids and their mycorrhizal fungi has been proposed to result in closely related orchids associating with more genetically similar OMF, this pattern has generally not been observed (e.g., Shefferson et al. 2007; Shefferson et al. 2010). For example, Otero et al. (2011) examined orchids in the Pterostylidinae and found that multiple orchid taxa specialized on particular clades of OMF, but there was little evidence of co-diversification. We found that more closely-related species within *Platanthera* generally did not associate with more similar OMF. The exception to this was within the two groups of closely-related species that regularly co-occur and hybridize (*P. blephariglottis*/*P. ciliaris*/*P. cristata*/*P. x bicolor*/*P. x canbyi* and *P. grandiflora*/*P. psycodes*/*P. shriveri*). Similar to what Otero et al. found, these clades of species associated with the same OMF, but all were relative generalists, rather than the specialists that Otero identified.

We found that only the most closely related species, those that both co-occurred and naturally hybridized, associated with similar OMF, suggesting that knowing the OMF associated with a closely related species will not generally be helpful in determining which OMF an orchid needs. The orange and white-fringed orchids (*P. blephariglottis*, *P. ciliaris*, *P. cristata*, and *P. chapmanii*) in section *Blephariglottis* are known to co-occur and form natural hybrids. The most commonly hybridizing among these species, *P. blephariglottis* and *P. ciliaris*, and their hybrid, *P. x bicolor*, had communities of OMF associates that almost completely overlapped. The OMF communities of *P. cristata* and *P. chapmanii*, which is thought to be a species that originated as a hybrid between *P. cristata* and *P. ciliaris*, also had considerable OMF community overlap, both with each other and, to a lesser extent, with *P. blephariglottis*, *P. ciliaris*, and *P. x bicolor*, with which they also co-occur and hybridize. It is notable, however, that *P. chapmanii*, which was not genetically distinguishable from *P. ciliaris* in Hartvig et al. (2026), did associate with distinct OMF, perhaps suggesting undetected differentiation. However, *P. integrilabia*, which is also in section *Blephariglottis* and is also thought to at least occasionally form hybrids with *P. ciliaris*, was very specialized with respect to its OMF (with *d’*=0.81, and an effective species number of only 2), and associated with OMF fungi that were distantly related to the core OMF taxa that associated with other orchid species in the section. There were two roots of *P. ciliaris* that were found to host the *P. integrilabia* fungi, notably in populations where it co-occurred with *P. integrilabia*, but *P. integrilabia* was not found to associate with any of the more common *P. ciliaris* OMF.

Similarly, the purple fringed orchids (*P. grandiflora*,* P. psycodes*,* P. shriveri*), which are also known to hybridize and were genetically indistinguishable throughout areas of overlapping ranges (Hartvig et al. 2026), had completely overlapping OMF (Fig. 1). However, *P. lacera*, which is also in section *Fimbriata* and is reported to hybridize with *P. grandiflora* and *P. psycodes*, was very specific in its OMF associations and associated with a different OMF OTU than the purple fringed species, similar to the pattern seen between *P. integrilabia* and the other orchids in section *Blephariglottis*. It is also interesting to note that *P. flava*, which consistently associated with OMF that overlapped with the purple fringed orchids, is not generally known to co-occur with those species, perhaps suggesting that other factors also influence distribution of these species.

Such overlapping fungi between hybrids, species of hybrid origin, and parent species, may drive, as well be driven by hybridization and backcrossing, by allowing continued co-occurrence of parents and hybrids. These species that shared OMF were also very closely related. In contrast, Esposito et al. (2016) found that two closely-related European *Platanthera* species that hybridized associated with partially overlapping but distinct OMF communities, while their putative hybrid associated with OMF that completely overlapped with that of the parent species from which it was genetically indistinguishable. Such overlapping OMF communities may suggest that speciation among these closely related orchids is incomplete, with species that are farther along in the speciation process being characterized by differences in OMF communities and habitats.

Other studies have also found that closely-related orchids associated with similar OMF and interpreted that as evidence that OMF did not play an immediate role in orchid speciation (Barrett et al. 2010; Waterman et al. 2011; but see Taylor et al. 2004). However, Waterman et al. also found shifts in OMF taxa among orchid clades at higher taxonomic levels both within and among orchid genera, perhaps suggesting that speciation may proceed initially as shifts in pollinators, followed later by shifts in OMF associations. Co-occurring, but distantly related species have often been found to associate with different OMF taxa, and this was predicted to promote co-occurrence by alleviating competition (Waterman et al. 2011; Jacquemyn et al. 2014; Mennicken et al. 2023). This may suggest a role for ongoing competition in the ecology of co-occurring orchids that could fuel further future differentiation.

We also found that some genetically distinct subgroups within orchid species associated with different OMF communities. For example, *P. blephariglottis*, *P. ciliaris*, and *P. x bicolor* populations along the coastal plain were genetically intermixed (Evans et al. 2023; Hartvig et al. 2026) and had strongly overlapping OMF, apparent as overlapping OTUs and inclusion within the same cluster in the modularity analysis. In contrast populations in the Appalachian Mountains, where *P. blephariglottis* and *P. x bicolor* do not occur, and in inland populations, where the species rarely overlap, associated with distinct OMF communities. The regional patterning in associated OMF could reflect genetic differences within the species, differences in the coastal vs. mountain, vs. inland ecosystems that support different OMF communities, or some combination of the two. We cannot tease these drivers apart without manipulative experiments that are explicitly designed to test this, so it remains an avenue for future research (e.g., Duffy et al. 2019). When populations were grouped by whether they were composed of a single species or multiple hybridizing species, rather than by genetically identified subgroups, there was no significant modularity.

The three purple-fringed species, *P. grandiflora*, *P. psycodes*, and *P. shriveri* completely shared OMF associates throughout their ranges. This included the northwest part of *P. psycodes’* range (Minnesota, Wisconsin, and Michigan), where *P. grandiflora* and *P. shriveri* do not occur. That was also the one part of *P. psycodes*’ range where Hartvig et al. found it to be genetically distinct from *P. grandiflora* and *P. shriveri*. That these geographically separated and genetically distinct populations nevertheless associated with the same OMF as in the rest of its range, might suggest that shared OMF associates could have driven species overlap and resulting genetic mixing, perhaps as a re-expansion from distinct glacial refugia, but this remains to be tested.

### Defining specificity

We used two methods to estimate the diversity of OMF each orchid associated with and used them to define specificity. The two estimates, *d’* and effective species number, were only roughly correlated. Effective species number is known to be strongly affected by the number of samples analyzed. Although *d’* is still affected when the number of samples collected is very small, it is somewhat more independent of sample size and may provide a better estimate of orchid specificity (Blütgen et al. 2006). Effective species number reflects the total number of OMF found in association with each orchid.

Specific and moderately specific orchids, those with high or moderately high *d’*, also had species accumulation curves that plateaued after fewer samples than generalized species. This likely indicates that identifying the OMF in association with more specialized orchids will be relatively achievable for conservation organizations. More generalized species will take more effort to identify the full range of their OMF associations, but it may be less critical to do so. The generalized species may be true generalists, in which case many different OMF may be sufficient to support these orchids. They could also be apparent generalists (sensu Shefferson et al. 2019), where some OMF may be critical for orchid growth and survival, with other OMF providing lesser or supplemental, or even no, support. In this case, it would be most important to identify the critical OMF, but the type of analysis presented here can only describe which OMF are associated with which orchid, not how they function.

### Geographic and genetic similarity

We predicted that geographically closer populations of an orchid would have more similar OMF communities than those that were farther apart. For most species, we found little evidence of a geographic pattern of genetic relatedness within dominant OMF genera. This was apparent as non-significant Mantel statistics for most species, although it is worth noting that our sample sizes were quite small for a Mantel analysis. With small sample sizes that were, necessarily, distributed where the species occurred and permits could be obtained, rather than in the ideal geographic dispersion, the Mantel tests that we conducted had low power to detect geographic patterns. There were significant geographic patterns in genetic relatedness of OMF associated with *P. cristata* and *P. x bicolor* that reflected genetic differences in the orchids, indicating some potential for geographic, or perhaps habitat-driven, distinctions in OMF communities (e.g., Jacquemyn et al. 2016). Regardless of whether closer sites have more similar OMF or if all sites incorporate similar OMF, it may still be preferred to focus sampling at nearby sites for OMF that are intended for restoration, reintroduction, or translocation efforts. Local OMF sourcing would ensure that fungi are locally adapted and could potentially colonize a target conservation site, minimizing the possibility of introducing a fungus that could have unintended consequences.

### Required sampling intensity

The number of samples that was required to adequately describe the OMF associated with each orchid varied considerably among species. For specialized orchids, only a few (3–5) samples in each of a few (3–5) sites was needed to identify the majority of associated OMF, and the primary OMF were generally identified in as few as two samples at each of two sites, while generalized orchids required many more samples, both within and among sites. It was also possible to distinguish specialized and generalized orchids with as few as 3–5 samples in 2–3 sites (see Fig. 2). 

Not all conservation efforts likely require a full assessment of the OMF associated with an orchid. Identifying the full range might be important for conservation of an orchid species across its entire range, but for other types of conservation efforts, much less might be needed. For example, it might be sufficient to collect root samples from 5 to 10 individuals in ~ 5 sites that are habitat-matched and geographically close to a target site.

Is it necessary to identify the full range of OMF for conservation purposes? Most likely, what is necessary is to identify the most important OMF. Future research will be required to determine which OMF are critical to orchid growth, reproduction, and persistence. For the *Platanthera* species in this study, there were often a few very common and many uncommon OMF taxa. With metabarcoding of all fungi in orchid roots it is common to identify diverse fungi in orchid roots, many of which are not OMF, some far more common and abundant than others. For example, Oja et al. (2017) found that there were a few consistently-present OMF taxa in roots of orchids that were sampled across multiple habitats, in addition to many uncommon habitat-specific taxa. Perhaps conservation efforts would be most fruitful if focused on the common OMF. However, making such a decision requires first knowing which OMF are consistently associated with an orchid species.

There is no consensus about how to identify which of the diverse OMF taxa identified in an orchid root are important or whether some taxa may be interchangeable. From the perspective of population persistence, the most critical OMF may be the ones that are needed for seed germination, but it is unclear whether these fungi are always present in the roots of mature orchids. Ventre-Lespiaucq et al. (2021) found that many orchids had different OMF communities at germination, seedling, and mature stages, suggesting both that the OMF needed for seed germination may not be among the fungi identified in mature orchid roots and that seed germination may not be sufficient to identify which fungi are needed to support mature orchids. It is reasonable to expect that fungi that are associated with orchid roots across multiple sites, years, and developmental stages are likely to be important to the orchid, and identifying these common fungi may require far less sampling effort than a full description of all OMF associated with an orchid.

### Application to conservation projects

It is widely understood that for orchids to persist in self-sustaining populations, appropriate OMF must be present and abundant. This means that effective orchid conservation requires inclusion of appropriate OMF, which must begin with identifying which OMF are needed by a target orchid. However, the amount of effort needed to determine which OMF orchids need is often unknown, which can be a significant impediment in conservation planning. In this study, we focused on species in *Platanthera*, and we found that determining which OMF associated with specific and moderately specific orchids required a limited effort, and was likely very manageable for conservation organizations. Our results demonstrate that it will be important to identify the OMF associated with each orchid species rather than trying to predict them based on proxies. While full description of the OMF associated with generalized orchids was more daunting, it may be that multiple OMF can support these orchids, in which case a full OMF description may be unnecessary. Additional research will be needed to determine the best way to identify OMF that are critical to orchid growth and reproduction, but the identification of OMF associated with each orchid provides a framework on which additional research can build.

## Supplementary Information

Below is the link to the electronic supplementary material.

Supplementary Material 1

Supplementary Material 2

Supplementary Material 3

Supplementary Material 4

Supplementary Material 5

## Data Availability

The sequence datasets generated during and/or analyzed during the current study are available in GenBank, [accession numbers PX605450-PX606138].
