# Supplementary material for: Mycorrhizal fungal associations of the fringed orchids (Platanthera) in the US, inter- and intra-species variation
Source: Biodivers Conserv. 2026 Jan 23;35(2):48. doi: 10.1007/s10531-025-03233-4 (PMC12827425; doi:10.1007/s10531-025-03233-4)
Supplement: Supplementary file 1 — Supplementary Material 1 [file 10531_2025_3233_MOESM1_ESM.pdf]

Figure S1: Phylogenetic estimation of relationships between OMF identified as A) *Ceratobasidium* and B) *Tulasnella*. Sample names match those in Table S1. Trees were constructed using FastTree 2.1.11 (fastest setting, GTR model, rate categories of sites = 20) in Geneious Prime to illustrate similarities between sequenced OMF. Numbers at nodes are Fasttree support values, which range from <0.01 (very weakly supported) to 1 (strongly supported). The scale bar at the bottom of each tree indicates expected number of substitutions.

Figure S1-A: *Ceratobasidium*

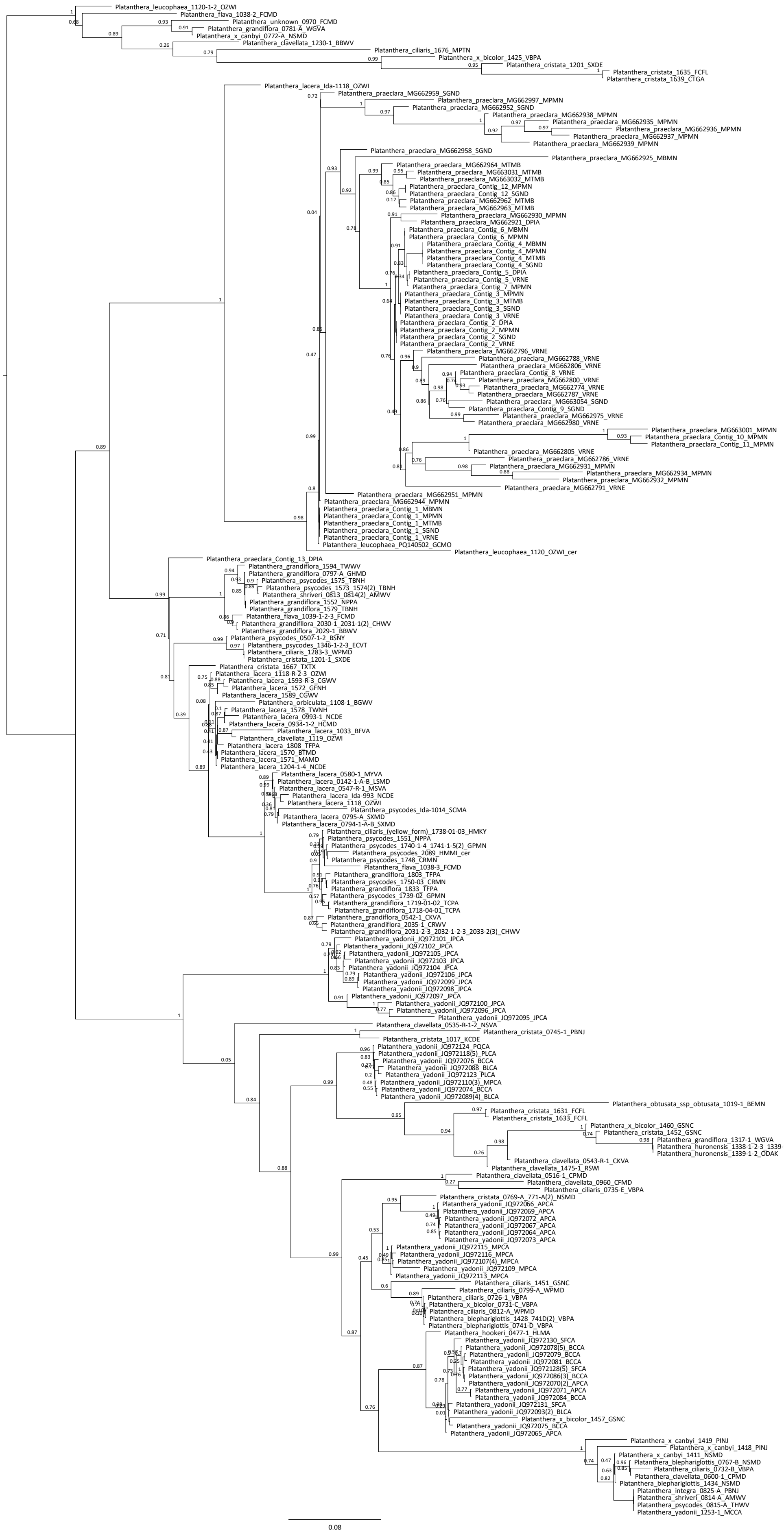

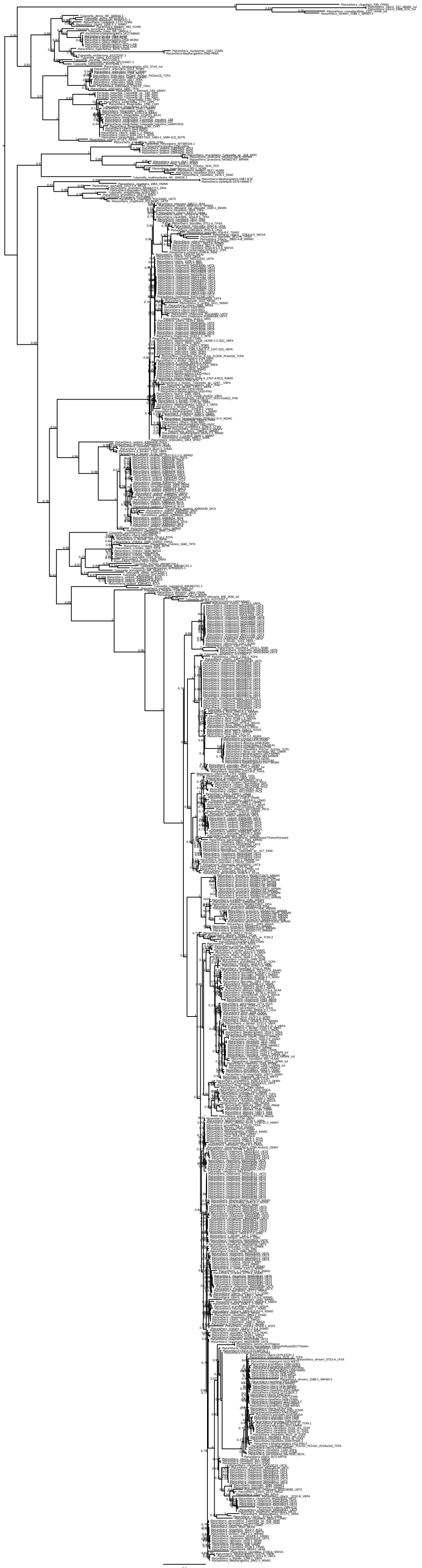

Figure S1-B. *Tulasnella*
