# Supplementary material for: Mycorrhizal fungal associations of the fringed orchids (Platanthera) in the US, inter- and intra-species variation
Source: Biodivers Conserv. 2026 Jan 23;35(2):48. doi: 10.1007/s10531-025-03233-4 (PMC12827425; doi:10.1007/s10531-025-03233-4)

Figure S2: The proportional (A) and numerical (B) representation of *Tulasnella* (blue bars), *Ceratobasidium* (green bars), and *Serendipita* (yellow bars) associated with orchid species in different subgenera and sections within *Platanthera*

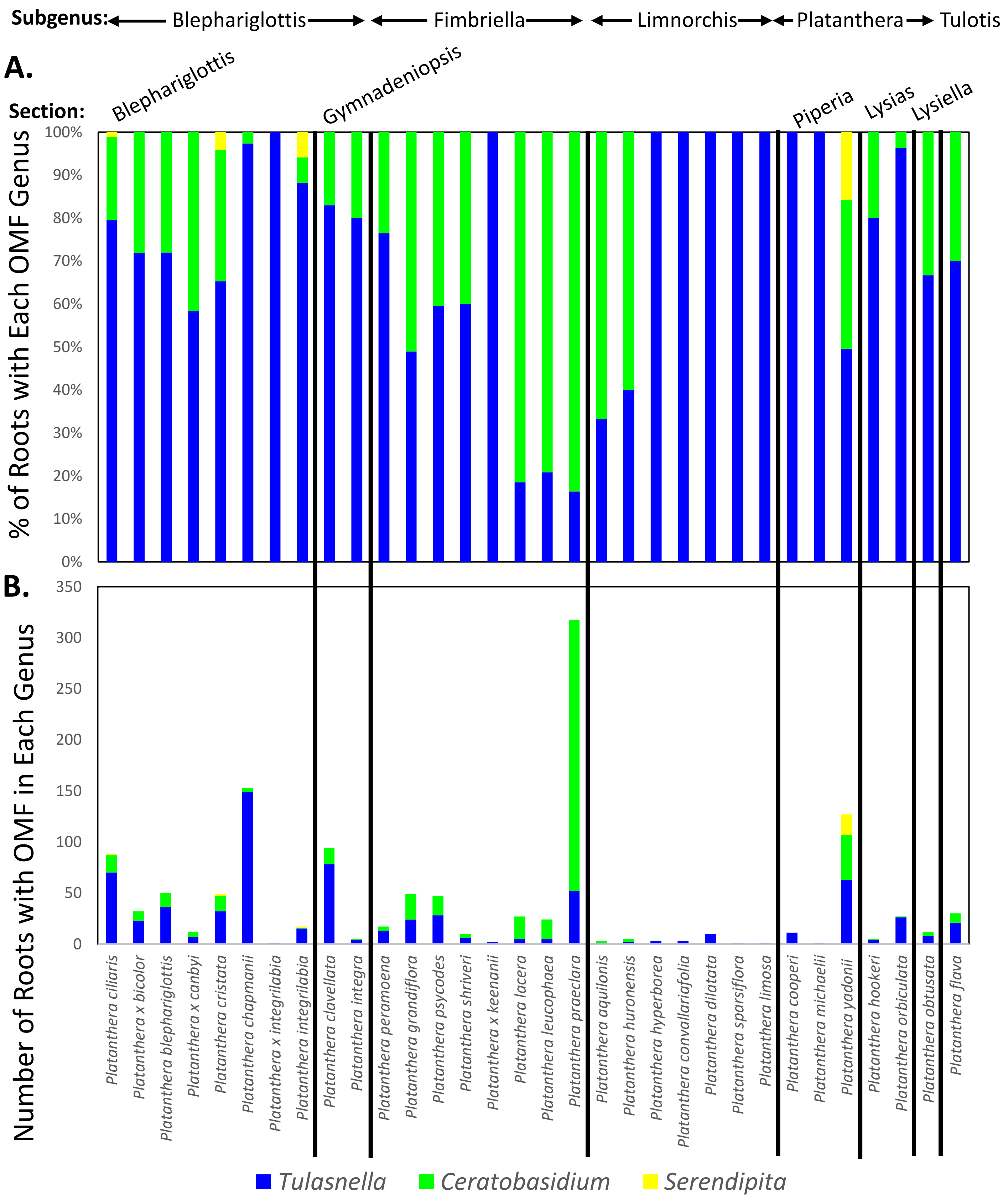

Supplement: Supplementary file 2 — Supplementary Material 2 [file 10531_2025_3233_MOESM2_ESM.pdf]
