# Supplementary material for: Mycorrhizal fungal associations of the fringed orchids (Platanthera) in the US, inter- and intra-species variation
Source: Biodivers Conserv. 2026 Jan 23;35(2):48. doi: 10.1007/s10531-025-03233-4 (PMC12827425; doi:10.1007/s10531-025-03233-4)
Supplement: Supplementary file 3 — Supplementary Material 3 [file 10531_2025_3233_MOESM3_ESM.pdf]

Figure S3: Ten modularity webs generated using ComputeModules with random seeds, showing the extent to which some taxa move among modules. Across the modularity webs, one cluster consistently has at least *P. blephariglottis*, *P. ciliaris*, and *P. x bicolor*. Nine of ten runs also included *P. x canbyi* in this module. The second consistent module included at least *P. grandiflora*, *P. psycodes*, *P. shriveri*, *P. peramoena*, and *P. flava*. Other modules were inconsistent and were rarely the same among runs.

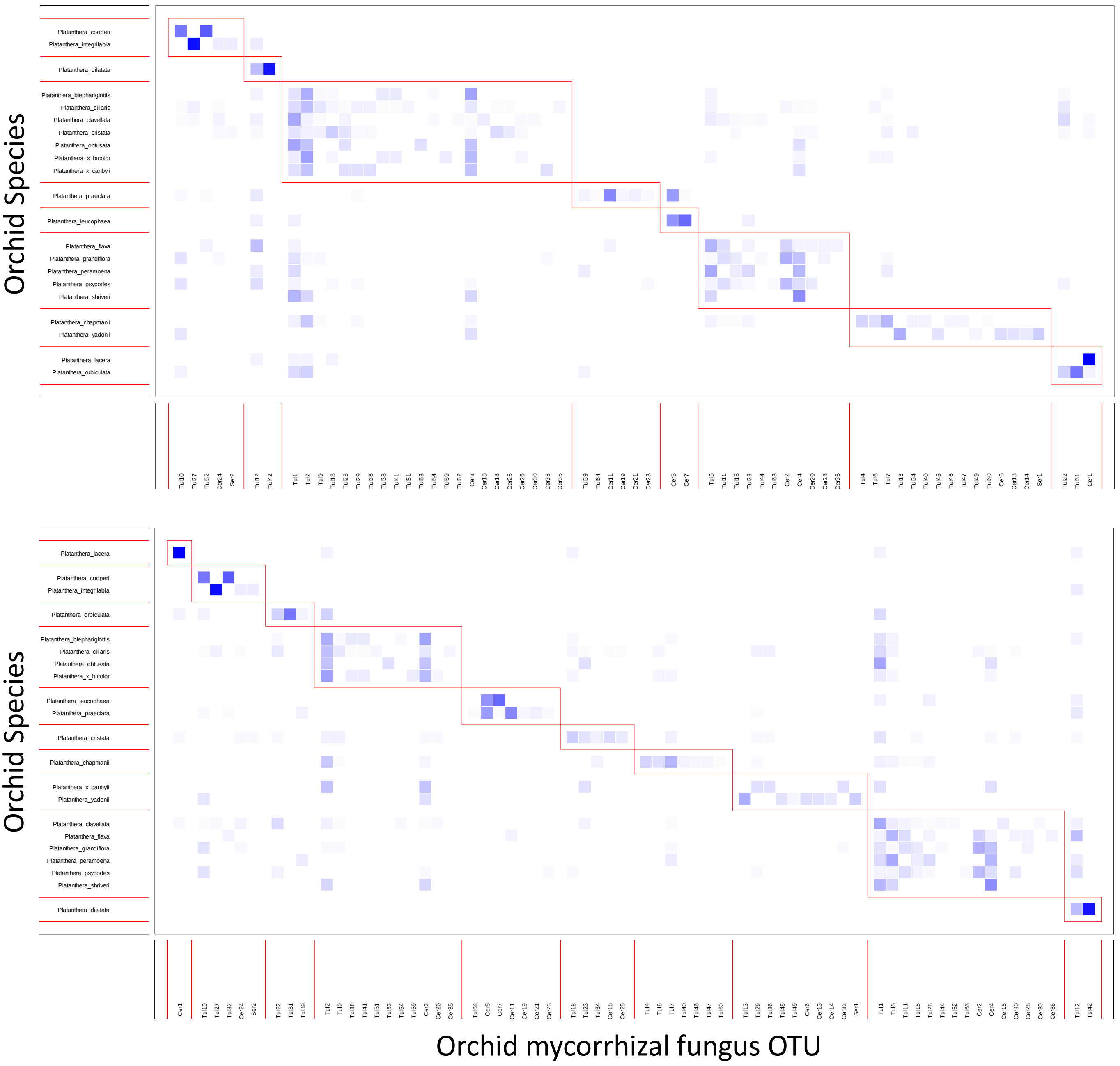

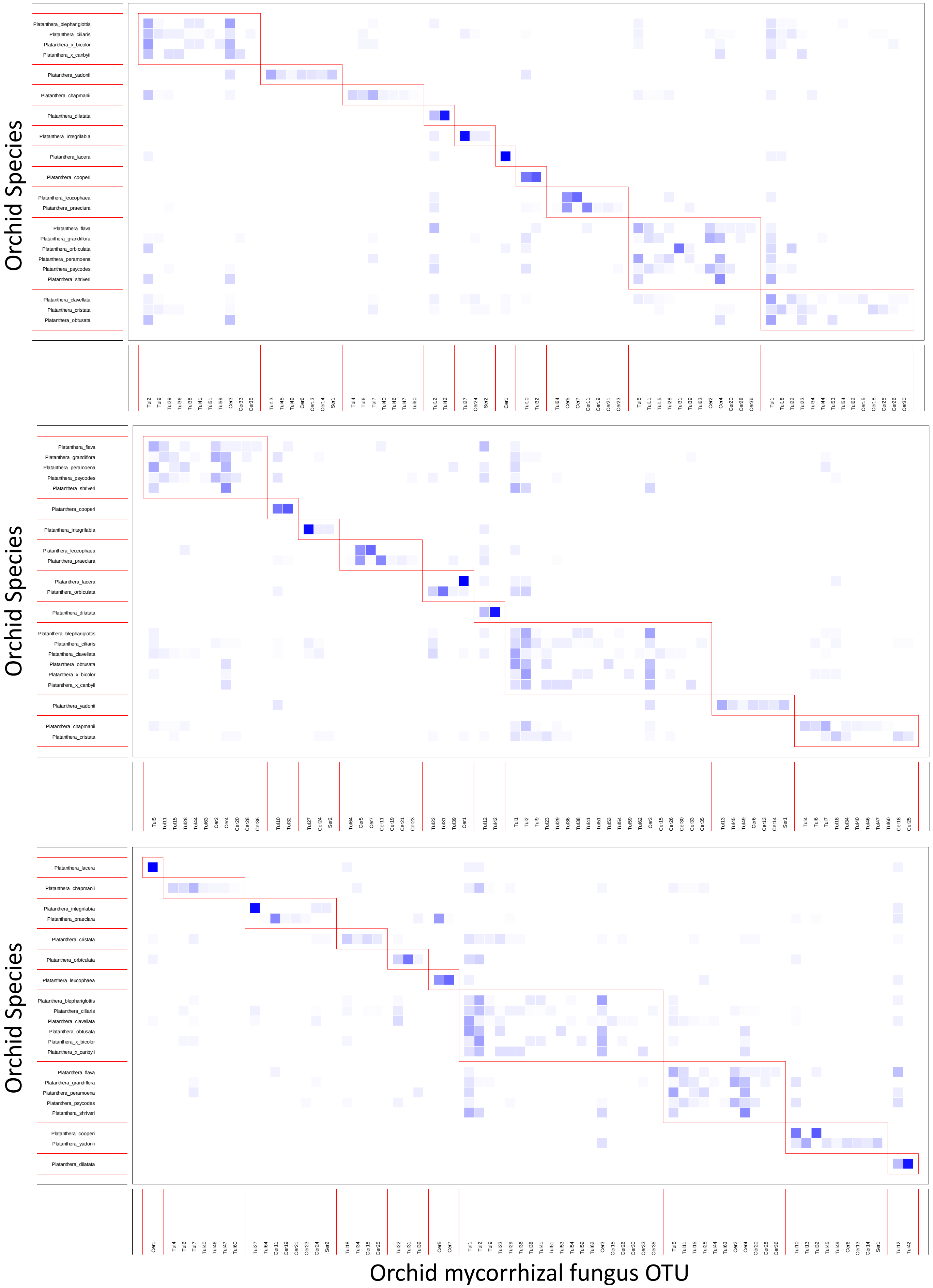
Orchid Species
Platanthera_blephariglottis
Platanthera_ciliaris
Platanthera_x_bicolor
Platanthera_x_canbyii
Platanthera_yadonii
Platanthera_chapmanii
Platanthera_dilatata
Platanthera_integrilabia
Platanthera_lacera
Platanthera_cooperi
Platanthera_leucophaea
Platanthera_praeclara
Platanthera_flava
Platanthera_grandiflora
Platanthera_orbiculata
Platanthera_peramoena
Platanthera_psycodes
Platanthera_shriveri
Platanthera_clavellata
Platanthera_cristata
Platanthera_obtusata
Tul2 Tul9 Tul29 Tul36 Tul38 Tul41 Tul51 Tul59 Cer3 Cer33 Cer35 Tul13 Tul45 Tul49 Cer6 Cer13 Cer14 Ser1 Tul4 Tul6 Tul7 Tul40 Tul46 Tul47 Tul60 Tul12 Tul42 Tul27 Cer24 Ser2 Cer1 Tul10 Tul32 Tul64 Cer5 Cer7 Cer11 Cer19 Cer21 Cer23 Tul5 Tul11 Tul15 Tul28 Tul31 Tul39 Tul63 Cer2 Cer4 Cer20 Cer28 Cer36 Tul1 Tul18 Tul22 Tul23 Tul34 Tul44 Tul53 Tul54 Tul62 Cer15 Cer18 Cer25 Cer26 Cer30
Orchid Species
Platanthera_flava
Platanthera_grandiflora
Platanthera_peramoena
Platanthera_psycodes
Platanthera_shriveri
Platanthera_cooperi
Platanthera_integrilabia
Platanthera_leucophaea
Platanthera_praeclara
Platanthera_lacera
Platanthera_orbiculata
Platanthera_dilatata
Platanthera_blephariglottis
Platanthera_ciliaris
Platanthera_clavellata
Platanthera_obtusata
Platanthera_x_bicolor
Platanthera_x_canbyii
Platanthera_yadonii
Platanthera_chapmanii
Platanthera_cristata
Tul5 Tul11 Tul15 Tul28 Tul44 Tul63 Cer2 Cer4 Cer20 Cer28 Cer36 Tul10 Tul32 Tul27 Cer24 Ser2 Tul64 Cer5 Cer7 Cer11 Cer19 Cer21 Cer23 Tul22 Tul31 Tul39 Cer1 Tul12 Tul42 Tul1 Tul2 Tul9 Tul23 Tul29 Tul36 Tul38 Tul41 Tul51 Tul53 Tul54 Tul59 Tul62 Cer3 Cer15 Cer26 Cer30 Cer33 Cer35 Tul13 Tul45 Tul49 Cer6 Cer13 Cer14 Ser1 Tul4 Tul6 Tul7 Tul18 Tul34 Tul40 Tul46 Tul47 Tul60 Cer18 Cer25
Orchid Species
Platanthera_lacera
Platanthera_chapmanii
Platanthera_integrilabia
Platanthera_praeclara
Platanthera_cristata
Platanthera_orbiculata
Platanthera_leucophaea
Platanthera_blephariglottis
Platanthera_ciliaris
Platanthera_clavellata
Platanthera_obtusata
Platanthera_x_bicolor
Platanthera_x_canbyii
Platanthera_flava
Platanthera_grandiflora
Platanthera_peramoena
Platanthera_psycodes
Platanthera_shriveri
Platanthera_cooperi
Platanthera_yadonii
Platanthera_dilatata
Cer1 Tul4 Tul6 Tul7 Tul40 Tul46 Tul47 Tul60 Tul27 Tul64 Cer11 Cer19 Cer21 Cer23 Cer24 Ser2 Tul18 Tul34 Cer18 Cer25 Tul22 Tul31 Tul39 Cer5 Cer7 Tul1 Tul2 Tul9 Tul23 Tul29 Tul36 Tul38 Tul41 Tul51 Tul53 Tul54 Tul59 Tul62 Cer3 Cer15 Cer26 Cer30 Cer33 Cer35 Tul5 Tul11 Tul15 Tul28 Tul44 Tul63 Cer2 Cer4 Cer20 Cer28 Cer36 Tul10 Tul13 Tul32 Tul45 Tul49 Cer6 Cer13 Cer14 Ser1 Tul12 Tul42
Orchid mycorrhizal fungus OTU

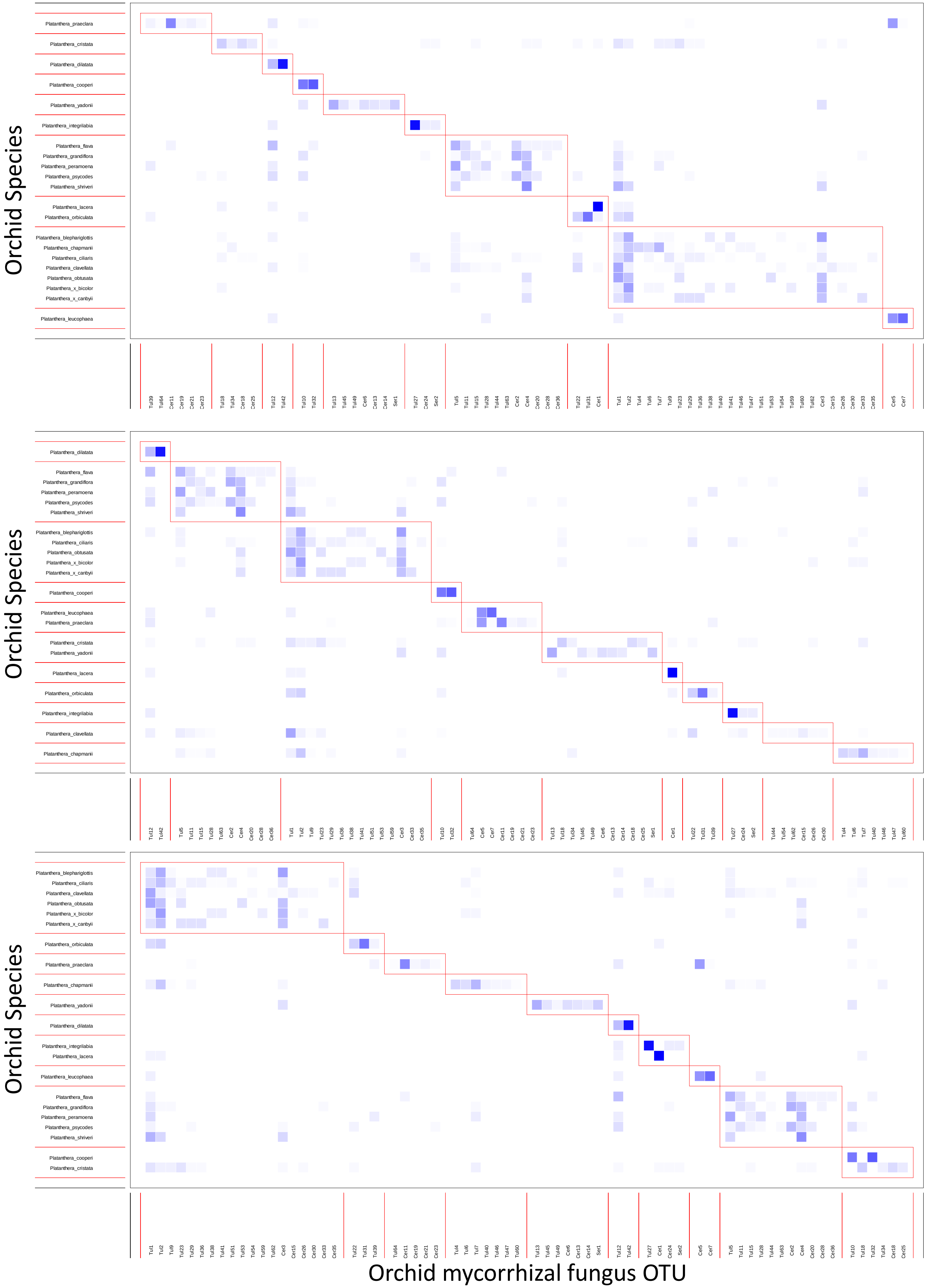
Orchid Species
Platanthera_praeclara
Platanthera_cristata
Platanthera_dilatata
Platanthera_cooperi
Platanthera_yadonii
Platanthera_integrilabia
Platanthera_flava
Platanthera_grandiflora
Platanthera_peramoena
Platanthera_psycodes
Platanthera_shriveri
Platanthera_lacera
Platanthera_orbiculata
Platanthera_blephariglottis
Platanthera_chapmanii
Platanthera_ciliaris
Platanthera_clavellata
Platanthera_obtusata
Platanthera_x_bicolor
Platanthera_x_canbyii
Platanthera_leucophaea
Orchid Species
Orchid Species
Orchid mycorrhizal fungus OTU

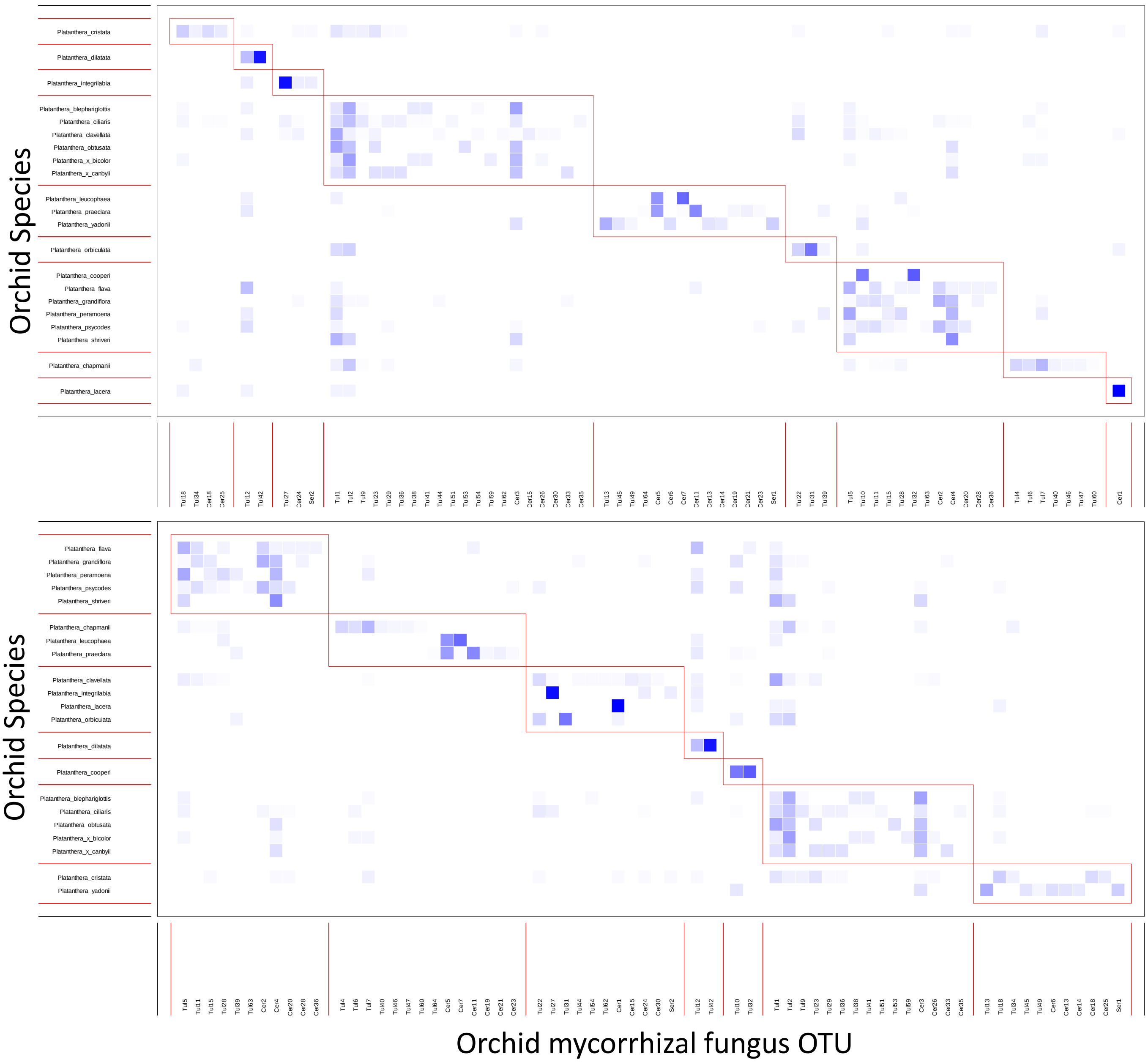
Orchid Species
Platanthera_cristata
Platanthera_dilatata
Platanthera_integrilabia
Platanthera_blephariglottis
Platanthera_ciliaris
Platanthera_clavellata
Platanthera_obtusata
Platanthera_x_bicolor
Platanthera_x_canbyii
Platanthera_leucophaea
Platanthera_praeclara
Platanthera_yadonii
Platanthera_orbiculata
Platanthera_cooperi
Platanthera_flava
Platanthera_grandiflora
Platanthera_peramoena
Platanthera_psycodes
Platanthera_shriveri
Platanthera_chapmanii
Platanthera_lacera
Tul18 Tul34 Cer18 Cer25 Tul12 Tul42 Tul27 Cer24 Ser2 Tul1 Tul2 Tul9 Tul23 Tul29 Tul36 Tul38 Tul41 Tul44 Tul51 Tul53 Tul54 Tul59 Tul62 Cer3 Cer15 Cer26 Cer30 Cer33 Cer35 Tul13 Tul45 Tul49 Tul64 Cer5 Cer6 Cer7 Cer11 Cer13 Cer14 Cer19 Cer21 Cer23 Ser1 Tul22 Tul31 Tul39 Tul5 Tul10 Tul11 Tul15 Tul28 Tul32 Tul63 Cer2 Cer4 Cer20 Cer28 Cer36 Tul4 Tul6 Tul7 Tul40 Tul46 Tul47 Tul60 Cer1
Orchid Species
Platanthera_flava
Platanthera_grandiflora
Platanthera_peramoena
Platanthera_psycodes
Platanthera_shriveri
Platanthera_chapmanii
Platanthera_leucophaea
Platanthera_praeclara
Platanthera_clavellata
Platanthera_integrilabia
Platanthera_lacera
Platanthera_orbiculata
Platanthera_dilatata
Platanthera_cooperi
Platanthera_blephariglottis
Platanthera_ciliaris
Platanthera_obtusata
Platanthera_x_bicolor
Platanthera_x_canbyii
Platanthera_cristata
Platanthera_yadonii
Tul5 Tul11 Tul15 Tul28 Tul39 Tul63 Cer2 Cer4 Cer20 Cer28 Cer36 Tul4 Tul6 Tul7 Tul40 Tul46 Tul47 Tul60 Tul64 Cer5 Cer7 Cer11 Cer19 Cer21 Cer23 Tul22 Tul27 Tul31 Tul44 Tul54 Tul62 Cer1 Cer15 Cer24 Cer30 Ser2 Tul12 Tul42 Tul10 Tul32 Tul1 Tul2 Tul9 Tul23 Tul29 Tul36 Tul38 Tul41 Tul51 Tul53 Tul59 Cer3 Cer26 Cer33 Cer35 Tul13 Tul18 Tul34 Tul45 Tul49 Cer6 Cer13 Cer14 Cer18 Cer25 Ser1
Orchid mycorrhizal fungus OTU
