# Supplementary material for: Mycorrhizal fungal associations of the fringed orchids (Platanthera) in the US, inter- and intra-species variation
Source: Biodivers Conserv. 2026 Jan 23;35(2):48. doi: 10.1007/s10531-025-03233-4 (PMC12827425; doi:10.1007/s10531-025-03233-4)
Supplement: Supplementary file 4 — Supplementary Material 4 [file 10531_2025_3233_MOESM4_ESM.pdf]

Table S1: Samples used in the current study. Sample Names are presented as the Species, Sample ID, and a 4 letter code for location. Columns indicate whether each site had one Platanthera species (Single) or had multiple species that might hybridize (Mixed). "Year" provides the year the orchid root was sampled, when available. Some sequences are from fungi isolated by collaborators, and year of isolation is not yet in our database. Latitude and Longitude are truncated to protect location. Cultures indicates the number of fungi that were cultured from pelotons in a root and sequenced using Sanger sequencing. SangerRoot indicates the number of sequences obtained using Sanger sequencing with *Tulasnella - Ceratobasidium* , and *Serendipita* PCR primers. MetaRoot indicates whether metabarcoding was conducted on the root remant. For each of the sequencing columns, the number in parentheses indicates the number of distinct OMF OTUs that were identified.

Species_Sample#_LocationCode	Single Species or Mixed	Year	latitude	longitude	Cultures	SangerRoot	MetaRoot
Platanthera_holochila_sp._330_XXHI	Single		not provided		1(1)		
Platanthera_holochila_sp._331_XXHI	Single		not provided		1(1)		
Platanthera_holochila_sp._332_XXHI	Single		not provided		1(1)		
Platanthera_aquilonis_1949_RSWI	Single	2023	45.1	-87.1		1(1)	
Platanthera_aquilonis_1950_RSWI	Single	2023	45.1	-87.1		1(1)	
Platanthera_aquilonis_2236_1_OWMN	Single		44.4	-93.9	1(1)		
Platanthera_blephariglottis_0642-1_NBMD	Mixed(cristata)	2004	38.3	-75.5	1(1)		
Platanthera_blephariglottis_0645R-1-2-4_NBMD	Mixed(cristata)	2004	38.3	-75.5	3(2)	1(1)	
Platanthera_blephariglottis_655_CCVA	Single	2004	38.9	-75.9	1(1)		
Platanthera_blephariglottis_0727-A-C-1_VBPA	Mixed(ciliaris)	2009	41.0	-76.0	3(2)		
Platanthera_blephariglottis_0734-1_VBPA	Mixed(ciliaris)	2009	41.0	-76.0	1(1)		
Platanthera_blephariglottis_0737-A_VBPA	Mixed(ciliaris)	2009	41.0	-76.0	1(1)		
Platanthera_blephariglottis_0741-D_VBPA	Mixed(ciliaris)	2009	41.0	-76.0	1(1)		
Platanthera_blephariglottis_0766-A_NBMD	Mixed(cristata)	2009	38.3	-75.5	1(1)		
Platanthera_blephariglottis_0767-A-B-D_NBMD	Mixed(cristata)	2009	38.3	-75.5	3(2)		
Platanthera_blephariglottis_0789R-A_MANC	Single	2009	35.9	-75.7	1(1)	1(1)	
Platanthera_blephariglottis_0790-A-C-D-H_MANC	Single	2009	35.9	-75.7	5(2)		
Platanthera_blephariglottis_1291-2_1_VBPA	Mixed(ciliaris)	2018	41.0	-76.0	2(1)		
Platanthera_blephariglottis_1305-2_1_NBMD	Mixed(cristata)	2018	38.3	-75.5	2(1)		
Platanthera_blephariglottis_1309_NBMD	Mixed(cristata)	2018	38.3	-75.5			1(1)
Platanthera_blephariglottis_1412_NBMD	Mixed(cristata)	2019	38.3	-75.5			1(2)
Platanthera_blephariglottis_1417_PINJ	Mixed(cristata)	2019	39.7	74.5			1(1)
Platanthera_blephariglottis_1420R-1to7_PINJ	Mixed(cristata)	2019	39.7	74.5	7(3)		
Platanthera_blephariglottis_1421_PINJ	Mixed(cristata)	2019	39.7	74.5			1(2)
Platanthera_blephariglottis_1423-2to4_PINJ	Mixed(cristata)	2019	39.7	74.5	3(2)		
Platanthera_blephariglottis_1428_VBPA	Mixed(ciliaris)	2019	41.0	-76.0		1(1)	
Platanthera_blephariglottis_1429R-1-2-3_VBPA	Mixed(ciliaris)	2019	41.0	-76.0	3(2)		
Platanthera_blephariglottis_1434_NBMD	Mixed(cristata)	2019	38.3	-75.5		1(1)	
Platanthera_blephariglottis_1837_MPNH	Single	2022	44.4	-71.5			1(2)
Platanthera_blephariglottis_1839_MPNH	Single	2022	44.4	-71.5			1(1)
Platanthera_blephariglottis_1840_CBNH	Single	2022	44.4	-71.5			1(1)
Platanthera_blephariglottis_1841_CBNH	Single	2022	44.4	-71.5			1(1)
Platanthera_blephariglottis_1913-1_PZPA	Mixed(ciliaris)	2023	39.9	-75.7	1(1)		
Platanthera_blephariglottis_1914-1_PZPA	Mixed(ciliaris)	2023	39.9	-75.7	1(1)		
Platanthera_blephariglottis_1915-1_PZPA	Mixed(ciliaris)	2023	39.9	-75.7	1(1)		
Platanthera_blephariglottis_1957_WOMI	Single	2023	45.6	-84.6			1(2)
Platanthera_blephariglottis_1958_WOMI	Single	2023	45.6	-84.6			1(2)
Platanthera_blephariglottis_1960_PRMI	Single	2023	45.2	-84.4			1(2)
Platanthera_blephariglottis_1961_1_2_3_4_PRMI	Single	2023	45.2	-84.4	4(1)	1(2)	
Platanthera_blephariglottis_1987_FMSC	Mixed(blephariglottis, cristata)	2023	33.2	-79.7			1(2)
Platanthera_blephariglottis_1993_FMSC	Mixed(blephariglottis, cristata)	2023	33.2	-79.7			1(2)
Platanthera_blephariglottis_2025_LLMI	Single	2023	44.7	-85.7			1(1)
Platanthera_blephariglottis_2026_LLMI	Single	2023	44.7	-85.7			1(1)
Platanthera_chapmanii_1443_ABGA	Single	2019	30.8	-82.2			1(1)
Platanthera_chapmanii_1444_ABGA	Single	2019	30.8	-82.2			1(2)
Platanthera_chapmanii_1610-1_1_JHTX	Single	2020	30.8	-94.5	1(1)		
Platanthera_chapmanii_1611-2-3_WRTX	Single	2020	30.8	-94.6	2(1)		
Platanthera_chapmanii_1613_WRTX	Single	2020	30.8	-94.6			1(2)
Platanthera_chapmanii_1614-3_JHTX	Single	2020	30.8	-94.5	1(1)		
Platanthera_chapmanii_1615-1_WRTX	Single	2020	30.8	-94.6	1(1)		
Platanthera_chapmanii_1630_REFL	Single	2020	30.8	-82.0			1(1)
Platanthera_chapmanii_1641-1_NRGA	Single	2020	31.0	-82.0	1(1)		
Platanthera_chapmanii_1643_NRGA	Single	2020	31.0	-82.0			1(1)
Platanthera_ciliaris_337_ABMD	Single	2001	39.1	-76.6	1(1)		
Platanthera_ciliaris_0555_NEVA	Single	2003	37.9	-78.9	1(1)		
Platanthera_ciliaris_0579R-1_AUVA	Single	2003	38.0	-78.9	1(1)	1(1)	
Platanthera_ciliaris_586_AUVA	Single	2003	38.2	-79.2	1(1)		
Platanthera_ciliaris_0587-R-1_AUVA	Single	2003	38.0	-78.9	1(1)	1(1)	
Platanthera_ciliaris_0592-R-1_CMVA	Single	2003	38.1	-78.8	1(1)	1(1)	
Platanthera_ciliaris_0714-1_ABMD	Single	2006	39.1	-76.6	1(1)		
Platanthera_ciliaris_0726-A-B_VBPA	Mixed(ciliaris)	2009	41.0	-76.0	2(1)		

Species_Sample#_LocationCode	Single Species or Mixed	Year	latitude	longitude	Cultures	SangerRoot	MetaRoot
Platanthera_ciliaris_0732-A-B_VBPA	Mixed(ciliaris)	2009	41.0	-76.0	2(2)		
Platanthera_ciliaris_0735-A-B-C-D-1_VBPA	Mixed(ciliaris)	2009	41.0	-76.0	5(2)		
Platanthera_ciliaris_0739-A-B-C_VBPA	Mixed(ciliaris)	2009	41.0	-76.0	3(3)		
Platanthera_ciliaris_0783R-A-D_WGVA	Single	2009	37.9	-79.0	2(2)	1(1)	
Platanthera_ciliaris_0799-A_WPMD	Single	2009	39.6	-77.5	1(1)		
Platanthera_ciliaris_0800-A_WPMD	Single	2009	39.6	-77.5	1(1)		
Platanthera_ciliaris_801_WPMD	Single	2009	39.6	-77.5			1(3)
Platanthera_ciliaris_0803-A_WPMD	Single	2009	39.6	-77.5	1(1)		
Platanthera_ciliaris_0805-A-B_WPMD	Single	2009	39.6	-77.5	2(2)		
Platanthera_ciliaris_0808-R-1-A_WPMD	Single	2009	39.6	-77.5	2(2)	1(1)	
Platanthera_ciliaris_0812R-A_WPMD	Single	2009	39.6	-77.5	1(1)	1(1)	
Platanthera_ciliaris_1045_1_WPMD	Single	2016	39.6	-77.5	1(1)		
Platanthera_ciliaris_1046_WPMD	Single	2016	39.6	-77.5			1(1)
Platanthera_ciliaris_1047_WPMD	Single	2016	39.6	-77.5			1(1)
Platanthera_ciliaris_1229_BBWV	Single	2017	39.1	-80.0			1(2)
Platanthera_ciliaris_1283-3_WPMD	Single	2018	39.6	-77.5	1(1)	1(1)	
Platanthera_ciliaris_1285_WPMD	Single	2018	39.6	-77.5			1(1)
Platanthera_ciliaris_1357-1_PZPA	Mixed(ciliaris)	2018	39.9	-75.7	1(1)		
Platanthera_ciliaris_1366-1_PZPA	Mixed(ciliaris)	2018	39.9	-75.7	1(1)		
Platanthera_ciliaris_1367-1_PZPA	Mixed(ciliaris)	2018	39.9	-75.7	1(1)		
Platanthera_ciliaris_1374_1426R-2to4_VBPA	Mixed(ciliaris)	2018	41.0	-76.0		1(1)	
Platanthera_ciliaris_1375-1_VBPA	Mixed(ciliaris)	2018	41.0	-76.0	1(1)		
Platanthera_ciliaris_1426R-1-2to4(2)_VBPA	Mixed(ciliaris)	2019	41.0	-76.0	4(2)	1(1)	
Platanthera_ciliaris_1436R-1-2-3_WPMD	Single	2019	39.6	-77.5	3(2)	1(1)	
Platanthera_ciliaris_1440-R-1-2-3-4_WPMD	Single	2019	39.6	-77.5	4(1)	1(1)	
Platanthera_ciliaris_1448_GSNC	Mixed(blephariglottis, cristata)	2019	34.1	-78.3			1(1)
Platanthera_ciliaris_1450_GSNC	Mixed(blephariglottis, cristata)	2019	34.1	-78.3			1(1)
Platanthera_ciliaris_1451-1-2-3_1_GSNC	Mixed(blephariglottis, cristata)	2019	34.1	-78.3	3(1)		1(1)
Platanthera_ciliaris_1456_GSNC	Mixed(blephariglottis, cristata)	2019	34.1	-78.3			1(1)
Platanthera_ciliaris_1516_WPMD	Single	2019	39.6	-77.5		1(2)	
Platanthera_ciliaris_1623R-1_BRFL	Single	2020	30.8	-86.9	1(1)		1(1)
Platanthera_ciliaris_1624_BRFL	Single	2020	30.8	-86.9			1(1)
Platanthera_ciliaris_1625-1_BRFL	Single	2020	30.9	-87.0	1(1)	1(1)	
Platanthera_ciliaris_1626_BRFL	Single	2020	30.8	-86.9			1(1)
Platanthera_ciliaris_1628-2_BRFL	Single	2020	30.8	-86.9	1(1)		
Platanthera_ciliaris_1629_BRFL	Single	2020	30.8	-86.9			1(1)
Platanthera_ciliaris_1648_MFGA	Mixed(cristata)	2020	31.9	-82.3			1(1)
Platanthera_ciliaris_1649-2_MFGA	Mixed(cristata)	2020	31.9	-82.3	1(1)		
Platanthera_ciliaris_1652_CBGA	Single	2020	32.2	-82.0			1(1)
Platanthera_ciliaris_1654_CBGA	Single	2020	32.2	-82.0			1(1)
Platanthera_ciliaris_1655-1_HWGA	Single	2020	34.7	-85.5	2(2)		
Platanthera_ciliaris_1657_HWGA	Single	2020	34.7	-85.5			1(2)
Platanthera_ciliaris_1659-1_SWGA	Single	2020	32.6	-84.4	1(1)		
Platanthera_ciliaris_1660-1_1_MMGA	Single	2020	34.6	-85.4	1(1)		
Platanthera_ciliaris_1661_MMGA	Single	2020	34.6	-85.4			1(1)
Platanthera_ciliaris_1662-2_MMGA	Single	2020	34.6	-85.4	1(1)		
Platanthera_ciliaris_1671_FMGA	Mixed(cristata)	2020	34.8	-84.7			1(1)
Platanthera_ciliaris_1672_FMGA	Mixed(cristata)	2020	34.8	-84.7			1(1)
Platanthera_ciliaris_1675_MPTN	Single	2020	35.7	-85.3			1(2)
Platanthera_ciliaris_1676_MPTN	Single	2020	35.8	-85.3			1(1)
Platanthera_ciliaris_1681_MBTN	Single	2020	35.7	-85.3			1(2)
Platanthera_ciliaris_1688-1_1_SGTN	Single	2020	35.5	-85.6	1(1)		
Platanthera_ciliaris_1694-1T1_1_BSTN	Single	2020	35.7	-85.2	2(2)		
Platanthera_ciliaris_1698-3_1_HMKY	Single	2020	37.2	-84.5	2(1)		
Platanthera_ciliaris_1699-2-3_HMKY	Single	2020	37.2	-84.5	2(1)		
Platanthera_ciliaris_1736-01-2_HMKY	Single	2021	37.2	-84.5	3(3)		
Platanthera_ciliaris_1738-01-03_HMKY	Single	2021	37.2	-84.5	2(2)		
Platanthera_ciliaris_1702_HMKY	Single	2020	37.2	-84.5			1(1)
Platanthera_ciliaris_1759-02_MLAL	Mixed(integrilabia)	2021	33.7	-85.8	1(1)		
Platanthera_ciliaris_1862_SFOH	Single	2022	38.7	-83.1			1(2)
Platanthera_ciliaris_1979_KTOH	Single	2023	41.6	-83.8			1(2)
Platanthera_ciliaris_1980_KTOH	Single	2023	41.6	-83.8			1(1)
Platanthera_ciliaris_1988_FMSC	Mixed(blephariglottis, cristata)	2023	33.2	-79.7			1(1)
Platanthera_ciliaris_1989_FMSC	Mixed(blephariglottis, cristata)	2023	33.2	-79.7			1(2)
Platanthera_ciliaris_1990_FMSC	Mixed(blephariglottis, cristata)	2023	33.2	-79.7			1(2)
Platanthera_clavellata_493_HLMA	Single	2002	42.6	-72.7	1(1)		1(2)
Platanthera_clavellata_0516-3_CPMD	Single	2003	38.4	-76.4	2(2)		
Platanthera_clavellata_0533-1_NSVA	Single	2003	38.0	-78.9	1(1)		
Platanthera_clavellata_0534R-1_NSVA	Single	2003	38.0	-78.9	1(1)	1(1)	

Species_Sample#_LocationCode	Single Species or Mixed	Year	latitude	longitude	Cultures	SangerRoot	MetaRoot
Platanthera_clavellata_0535-R-1-2_NSVA	Single	2003	38.0	-78.9	2(2)	1(1)	
Platanthera_clavellata_0543-R-1_CKVA	Single	2003	38.2	-79.3	1(1)	1(2)	
Platanthera_clavellata_0591-R-1_CMVA	Single	2003	38.1	-78.8	1(1)	1(1)	
Platanthera_clavellata_599_CPMD	Single	2003	38.4	-76.4			1(1)
Platanthera_clavellata_0600_CPMD	Single	2003	38.4	-76.4	1(1)		1(1)
Platanthera_clavellata_601_CPMD	Single	2003	38.4	-76.4			1(1)
Platanthera_clavellata_0602-1_CPMD	Single	2003	38.4	-76.4	1(1)		
Platanthera_clavellata_0604-1_CPMD	Single	2003	38.4	-76.4	1(1)		
Platanthera_clavellata_606_CPMD	Single	2003	38.4	-76.4			1(1)
Platanthera_clavellata_0607-1_CPMD	Single	2003	38.4	-76.4	1(1)		
Platanthera_clavellata_0609_CPMD	Single	2003	38.4	-76.4			1(2)
Platanthera_clavellata_0610-1_CPMD	Single	2003	38.4	-76.4	1(1)		
Platanthera_clavellata_611_CPMD	Single	2003	38.4	-76.4			1(1)
Platanthera_clavellata_0742-A_PBNJ	Single	2009	39.8	-74.9	1(1)		
Platanthera_clavellata_0749-A-C_MDVA	Single	2009	38.4	-79.6	2(2)		
Platanthera_clavellata_0750-A-1-3-4_MDVA	Single	2009	38.4	-79.6	4(3)		
Platanthera_clavellata_0778-1_WGVA	Single	2009	37.9	-79.0	1(1)		
Platanthera_clavellata_0779-A_WGVA	Single	2009	37.9	-79.0	1(1)		
Platanthera_clavellata_0782-A_WGVA	Single	2009	37.9	-79.0	1(1)		
Platanthera_clavellata_0785A-C_CBWV	Single	2009	38.6	-79.9	2(2)		
Platanthera_clavellata_0786-AtoD_CBWV	Single	2009	38.6	-79.9	4(1)		
Platanthera_clavellata_0827-A_PBNJ	Single	2010	39.8	74.9	1(1)		
Platanthera_clavellata_0829-B_PBNJ	Single	2010	39.8	74.9	1(1)		
Platanthera_clavellata_0959-R-1_CFMD	Single	2016	38.3	-75.5	1(1)	1(1)	
Platanthera_clavellata_0960_CFMD	Single	2016	38.3	-75.5			1(1)
Platanthera_clavellata_0961-1_CFMD	Single	2016	38.3	-75.5	1(1)	1(1)	
Platanthera_clavellata_0962-1_CFMD	Single	2016	38.3	-75.5	1(1)		
Platanthera_clavellata_1043R_FCMD1	Single	2017	39.6	-77.5		1(2)	
Platanthera_clavellata_1119_OZWI	Single	2017	43.4	-88.0			1(1)
Platanthera_clavellata_1149_SOPA	Single	2017	40.0	-79.1			1(2)
Platanthera_clavellata_1172_FCMA	Single	2017	42.5	-72.6			1(2)
Platanthera_clavellata_1218_CLME	Single	2017	43.7	-70.3			1(2)
Platanthera_clavellata_1219_CUME	Single	2017	43.7	-70.3			1(2)
Platanthera_clavellata_1226-2-4_BBWV	Single	2017	39.3	-79.9	3(2)		
Platanthera_clavellata_1230-1_BBWV	Single	2017	39.3	-79.9	1(1)	1(1)	
Platanthera_clavellata_1437-R-1-1-3_WPMD	Single	2019	39.6	-77.5	2(1)	1(2)	
Platanthera_clavellata_1441_WPMD	Single	2019	39.6	-77.5			1(1)
Platanthera_clavellata_1442_WPMD	Single	2019	39.6	-77.5			1(2)
Platanthera_clavellata_1474-1_RSWI	Single	2019	45.1	-86.9	1(1)		
Platanthera_clavellata_1475-1_RSWI	Single	2019	45.1	-86.9	1(1)		
Platanthera_clavellata_1476-1_RSWI	Single	2019	45.1	-86.9	1(1)		
Platanthera_clavellata_1518_WPMD	Single	2019	39.6	-77.5		1(2)	
Platanthera_clavellata_1606_CFMN	Single	2020	47.6	-93.9			1(2)
Platanthera_clavellata_1607_CFMN	Single	2020	47.6	-93.9			1(1)
Platanthera_clavellata_1608_CFMN	Single	2020	47.6	-93.9			1(1)
Platanthera_clavellata_1814-R-1_TFPA	Single	2022	41.7	-77.2	1(1)		
Platanthera_clavellata_1815_TFPA	Single	2022	41.7	-77.2			1(2)
Platanthera_clavellata_1825_TFPA	Single	2022	41.7	-77.2			1(2)
Platanthera_clavellata_1829_TFPA	Single	2022	41.7	-77.2			1(1)
Platanthera_clavellata_1873_TFPA	Single	2022	41.7	-77.2			1(1)
Platanthera_clavellata_1875_TFPA	Single	2022	41.7	-77.2			1(1)
Platanthera_clavellata_1951-1_IMMI	Single	2023	45.9	-88.0	1(1)		
Platanthera_clavellata_1952_IMMI	Single	2023	45.9	-88.0			1(1)
Platanthera_clavellata_1953_IMMI	Single	2023	45.9	-88.0			1(2)
Platanthera_clavellata_2076_HMMI	Single	2023	46.9	-87.8			1(2)
Platanthera_clavellata_2083_HMMI	Single	2023	46.9	-87.8			1(2)
Platanthera_clavellata_2100_HMMI	Single	2023	46.9	-87.8			1(2)
Platanthera_clavellata_2118-1-2-3-4_ORMN	Single		44.9	-93.4	4(1)		
Platanthera_clavellata_2124-1-2_ORMN	Single		44.9	-93.4	2(1)		
Platanthera_clavellata_2244-2_ORMN	Single		44.9	-93.4	1(1)		
Platanthera_clavellata_Pc1or_TCPA	Single	2022	41.7	-77.2			1(1)
Platanthera_clavellata_Pc2or_TCPA	Single	2022	41.7	-77.2			1(1)
Platanthera_clavellata_Pc3OR_TCPA	Single	2022	41.7	-77.2			1(1)
Platanthera_clavellata_PC4or_TCPA	Single	2022	41.7	-77.2			1(1)
Platanthera_clavellata_PcD1_or_TCPA	Single	2022	41.7	-77.2			1(1)
Platanthera_clavellata_PCD2or_TCPA	Single	2022	41.7	-77.2			1(1)
Platanthera_clavellata_PcD3_or_TCPA	Single	2022	41.7	-77.2			1(1)
Platanthera_clavellata_PcD4_ora_TCPA	Single	2022	41.7	-77.2			1(1)
Platanthera_clavellata_PCD5_or_TCPA	Single	2022	41.7	-77.2			1(1)

Species_Sample#_LocationCode	Single Species or Mixed	Year	latitude	longitude	Cultures	SangerRoot	MetaRoot
Platanthera_clavellata_0970_FCMD	Single	2016	39.6	-77.5			1(1)
Platanthera_convallariafolia_2008_PMAK	Single	2023	59.6	-151.6			1(1)
Platanthera_convallariafolia_2013_PMAK	Single	2023	59.6	-151.6			1(1)
Platanthera_convallariifolia_2257_AHAK	Single	2023	59.6	-151.5			1(1)
Platanthera_cristata_0745-1_PBNJ	Mixed(ciliaris)	2009	39.8	-74.9	1(1)		
Platanthera_cristata_0746-B_PBNJ	Mixed(ciliaris)	2009	39.8	-74.9	1(1)		
Platanthera_cristata_0769-A_NBMD	Mixed(ciliaris)	2009	38.3	-75.5	1(1)		
Platanthera_cristata_0770-A_NBMD	Mixed(ciliaris)	2009	38.3	-75.5	1(1)		
Platanthera_cristata_0771-A-B_NBMD	Mixed(ciliaris)	2009	38.3	-75.5	2(2)		
Platanthera_cristata_1016R-1_KCDE	Single	2016	39.0	-75.4	1(1)	1(2)	
Platanthera_cristata_1017_KCDE	Single	2016	39.0	-75.4			1(2)
Platanthera_cristata_1200_SXDE	Single	2017	39.0	-75.4			1(2)
Platanthera_cristata_1201-R-1_SXDE	Single	2017	39.0	-75.4	1(1)	1(2)	
Platanthera_cristata_1273_SXDE	Single	2018	39.0	-75.4			1(2)
Platanthera_cristata_1306_MDMD	Single	2018	38.3	-75.5			1(2)
Platanthera_cristata_1307_MDMD	Single	2018	38.3	-75.5			1(1)
Platanthera_cristata_1409_NBMD	Mixed(ciliaris)	2019	38.3	-75.5			1(2)
Platanthera_cristata_1430-1-2-3-4_NBMD	Mixed(ciliaris)	2019	38.3	-75.5	4(2)		
Platanthera_cristata_1431_NBMD	Mixed(ciliaris)	2019	38.3	-75.5			1(1)
Platanthera_cristata_1432_NBMD	Mixed(ciliaris)	2019	38.3	-75.5			1(1)
Platanthera_cristata_1433-R-1-2-3-4_NBMD	Mixed(ciliaris)	2019	38.3	-75.5	4(1)	1(1)	
Platanthera_cristata_1446-1_GSNC	Mixed(blephariglottis, ciliaris)	2019	34.1	-78.3	1(1)		
Platanthera_cristata_1447-1_GSNC	Mixed(blephariglottis, ciliaris)	2019	34.1	-78.3	1(1)		
Platanthera_cristata_1449_GSNC	Mixed(blephariglottis, ciliaris)	2019	34.1	-78.3			1(1)
Platanthera_cristata_1452-R-1_GSNC	Mixed(blephariglottis, ciliaris)	2019	34.1	-78.3	1(1)	1(2)	
Platanthera_cristata_1620-1_BRFL	Single	2020	30.9	-86.6	1(1)		
Platanthera_cristata_1621R-2_BRFL	Single	2020	30.9	-86.6	1(1)	1(1)	
Platanthera_cristata_1631_FCFL	Single	2020	30.5	-81.8			1(2)
Platanthera_cristata_1633_FCFL	Single	2020	30.5	-81.8			1(1)
Platanthera_cristata_1635_FCFL	Single	2020	30.6	-81.8			1(1)
Platanthera_cristata_1638_CTGA	Single	2020	30.9	-81.7			1(1)
Platanthera_cristata_1639_CTGA	Single	2020	30.9	-81.7			1(1)
Platanthera_cristata_1644_MFGA	Mixed(ciliaris)	2020	31.9	-82.3			1(1)
Platanthera_cristata_1645_MFGA	Mixed(ciliaris)	2020	31.9	-82.3			1(1)
Platanthera_cristata_1646_MFGA	Mixed(ciliaris)	2020	31.9	-82.3			1(1)
Platanthera_cristata_1666_TXTX	Single	2020	31.5	-93.9			1(1)
Platanthera_cristata_1667_TXTX	Single	2020	31.5	-93.9			1(2)
Platanthera_cristata_1668_FMGA	Mixed(ciliaris)	2020	34.8	-84.7			1(1)
Platanthera_cristata_1669_FMGA	Mixed(ciliaris)	2020	34.8	-84.7			1(1)
Platanthera_cristata_1680_MBTN	Single	2020	35.8	-85.3			1(1)
Platanthera_cristata_1689_SGTN	Mixed(integrilabia)	2020	35.5	-85.6			1(1)
Platanthera_cristata_1695_BFTN	Single	2020	35.7	-85.2			1(1)
Platanthera_cristata_1696_BFTN	Single	2020	35.7	-85.2			1(2)
Platanthera_cristata_1985_FMSC	Mixed	2023	33.2	-79.7			1(2)
Platanthera_dilatata_0679-1_MPTN	Single	2005	59.6	-151.2	1(1)		
Platanthera_dilatata_0680-1-2-3-4_HCAK	Single	2005	59.6	-151.2	4(1)	1(1)	
Platanthera_dilatata_0682-1_HCAK	Single	2005	59.6	-151.2	2(1)		
Platanthera_dilatata_1481-1_BIAK	Single	2018	57.1	-135.3	1(1)		
Platanthera_dilatata_1482-1_DIAK	Single	2018	58.3	-134.5	1(1)		
Platanthera_dilatata_1584_TWNH	Single	2020	43.9	-71.3			1(1)
Platanthera_dilatata_1948_RSWI	Single	2023	45.1	-86.9			1(1)
Platanthera_dilatata_2009_PMAK	Single	2023	59.6	-151.5			1(1)
Platanthera_dilatata_2015_PMAK	Single	2023	59.6	-151.5			1(1)
Platanthera_flava_0480-1_LHMA	Single	2002	42.4	-73.3	1(1)		
Platanthera_flava_0492-2_HLMA	Single	2002	42.6	-72.7	1(1)		
Platanthera_flava_0546R-1-2_MSVA	Single	2003	38.1	-78.8	2(1)	1(1)	
Platanthera_flava_0553-R-2_MSVA	Single	2003	38.1	-78.8	1(1)	1(1)	
Platanthera_flava_0594-1-2_OLVA	Single	2003	37.4	-79.6	2(1)	1(1)	
Platanthera_flava_0747-1_MDVA	Single	2009	38.4	-79.6	2(2)		
Platanthera_flava_0748-1-2_MDVA	Single	2009	38.4	-79.6	2(1)		
Platanthera_flava_0764-A-B_PHWI	Single	2009	43.4	-89.9	2(2)		
Platanthera_flava_0980-1_MNMN	Single	2016	47.5	-93.3	1(1)		
Platanthera_flava_0981-R-1-2_MNMN	Single	2016	47.5	-93.3	2(1)	1(2)	
Platanthera_flava_0982_MNMN	Single	2016	47.5	-93.3	1(1)		
Platanthera_flava_1038R-1_FCMD	Single	2016	39.6	-77.5	1(1)	1(2)	
Platanthera_flava_1039-1-2-3_FCMD	Single	2016	39.6	-77.5	3(1)		
Platanthera_flava_1102_TCMD	Single	2017	38.8	-76.1			1(1)
Platanthera_flava_1280_NCDE	Single	2018	39.7	-75.6			1(2)
Platanthera_flava_1497_MDMD	Single	2019	39.6	-77.5			1(1)

Species_Sample#_LocationCode	Single Species or Mixed	Year	latitude	longitude	Cultures	SangerRoot	MetaRoot
Platanthera_flava_1576-1-2_GFNH	Single	2020	43.5	-71.4	2(1)		
Platanthera_flava_1710_LCIL	Single	2021	42.2	-87.8			1(1)
Platanthera_flava_1967-1-2_SRIN	Single	2023	41.1	-87.5	2(1)		
Platanthera_flava_1968_SRIN	Single	2023	41.1	-87.5			1(1)
Platanthera_flava_2088_HMMI	Single	2024	46.9	-87.8			1(2)
Platanthera_flava_2092_HMMI	Single	2024	46.9	-87.8			1(2)
Platanthera_grandiflora_0527-R-1-2_NSVA	Single	2003	38.0	-78.9	2(2)		
Platanthera_grandiflora_0529-R-1-2_NSVA	Single	2003	38.0	-78.9	2(1)		
Platanthera_grandiflora_0542-1_CKVA	Single	2003	38.2	-79.3	1(1)	1(1)	
Platanthera_grandiflora_0777-B_WGVA	Single	2009	37.9	-79.0	1(1)		
Platanthera_grandiflora_0780-A_WGVA	Single	2009	37.9	-79.0	1(1)		
Platanthera_grandiflora_0781-A_WGVA	Single	2009	37.9	-79.0	1(1)		
Platanthera_grandiflora_0788-A_CTWV	Single	2009	38.8	-80.0	1(1)		
Platanthera_grandiflora_0797-A_GHMD	Single	2018	36.6	-77.5	2(1)		
Platanthera_grandiflora_1317-1_WGVA	Single	2018	37.9	-79.0	1(1)	1(2)	
Platanthera_grandiflora_1323_CRWV	Single	2018	38.7	-80.9			1(2)
Platanthera_grandiflora_1513_FCMD	Single	2019	39.6	-77.5			1(2)
Platanthera_grandiflora_1514_FCMD	Single	2019	39.6	-77.5			1(1)
Platanthera_grandiflora_1552_NPPA	Mixed(psycodes)	2019	41.1	-75.9			1(1)
Platanthera_grandiflora_1555_HGGA	Single	2019	34.8	-84.5			1(1)
Platanthera_grandiflora_1556_HGGA	Single	2019	34.8	-84.5			1(1)
Platanthera_grandiflora_1579_TBNH	Single	2019	43.7	-71.3			1(2)
Platanthera_grandiflora_1580_CHNH	Mixed(psycodes)	2019	43.9	-71.2			1(1)
Platanthera_grandiflora_1582_CHNH	Mixed(psycodes)	2019	43.9	-71.2			1(1)
Platanthera_grandiflora_1585_WRWV	Mixed(shriveri)	2019	38.2	-80.2			1(1)
Platanthera_grandiflora_1586_WRWV	Mixed(shriveri)	2019	38.2	-80.2			1(1)
Platanthera_grandiflora_1588_WRWV	Mixed(shriveri)	2019	38.2	-80.2			1(1)
Platanthera_grandiflora_1590_CGWV	Single	2019	38.2	-80.3			1(1)
Platanthera_grandiflora_1594_TWWV	Single	2019	38.6	-79.7			1(1)
Platanthera_grandiflora_1718-01_TCPA	Single	2021	41.6	-77.5	1(2)		
Platanthera_grandiflora_1719-01_TCPA	Single	2021	41.6	-77.5	1(2)		
Platanthera_grandiflora_1803_TCPA	Single	2022	41.6	-77.5	1(1)		1(2)
Platanthera_grandiflora_1833_TCPA	Single	2022	41.6	-77.5			1(2)
Platanthera_grandiflora_1878_TCPA	Single	2022	41.6	-77.5			1(1)
Platanthera_grandiflora_2027-1-2-3_BAWV	Single	2022	38.5	-79.9	3(1)		
Platanthera_grandiflora_2028-1_BBWV	Single	2022	39.1	-79.6	1(1)		
Platanthera_grandiflora_2029-1_BBWV	Single	2022	39.1	-79.6	1(1)		
Platanthera_grandiflora_2030-1_CHWV	Mixed(shriveri)	2022	38.6	-79.9	1(1)		
Platanthera_grandiflora_2031-1_CHWV	Mixed(shriveri)	2022	38.6	-79.8	1(1)		
Platanthera_grandiflora_2032-1-2-3_CHWV	Mixed(shriveri)	2022	38.6	-79.8	3(1)		
Platanthera_grandiflora_2033-1_CHWV	Mixed(shriveri)	2022	38.6	-79.9	1(1)		
Platanthera_grandiflora_2034-1-2_CRWV	Mixed(shriveri)	2022	38.3	-80.5	2(1)		
Platanthera_grandiflora_2035-1_CRWV	Mixed(shriveri)	2022	38.3	-80.5	1(1)		
Platanthera_grandiflora_2036-1-2-3-4-5_SKWV	Single	2022	38.7	-79.5	5(1)		
Platanthera_grandiflora_2038-1-2_SKWV	Single	2022	38.7	-79.5	2(1)		
Platanthera_grandiflora_PG1_or_TCPA	Single	2022	41.6	-77.5			1(2)
Platanthera_grandiflora_PG3_or_TCPA_1	Single	2022	41.6	-77.5			1(2)
Platanthera_grandiflora_1317-1_WGVA	Single	2018	37.9	-78.9	1(1)	1(2)	
Platanthera_hookeri_0477-1_HLMA	Single	2002	42.6	-72.7	1(1)	1(1)	
Platanthera_hookeri_0483_HLMA	Single	2002	42.6	-72.7			1(1)
Platanthera_hookeri_1537-2_BSMN	Single	2020	47.5	-93.3	1(1)		
Platanthera_hookeri_1540_BSMN	Single	2020	47.5	-93.3			1(1)
Platanthera_hookeri_2064_BSMN	Single		47.5	-93.3	1(1)		
Platanthera_huronensis_1021_CCMN	Single	2016	47.2	-94.2			1(1)
Platanthera_huronensis_1338-1-2-3_ODAK	Single	2018	59.6	-151.5	3(1)		
Platanthera_huronensis_1339-1-2_ODAK	Single	2018	59.6	-151.5	2(1)		
Platanthera_huronensis_2061_1_CRMN	Single		47.7	-90.5	1(1)		
Platanthera_hyperborea_0478_HLMA	Single	2002	42.6	-72.7			1(1)
Platanthera_hyperborea_0859-1_AKAK	Single	2013	59.6	-151.2	1(1)		
Platanthera_hyperborea_1765-1_HOAK	Single	2021	59.6	-151.5	1(1)		
Platanthera_integra_0743-1-A_PBNJ	Single	2009	39.8	-74.9	2(1)		
Platanthera_integra_0825-A_PBNJ	Single	2010	39.8	-74.9	2(2)		
Platanthera_integra_1454-R-1-2_GSNC	Single	2019	34.1	-78.3	2(1)	1(1)	
Platanthera_integra_1455-R-1_GSNC	Single	2019	34.1	-78.3	1(1)	1(1)	
Platanthera_integrilabia_1486-1_CPKY	Single	2020	37.5	-83.0	1(1)		
Platanthera_integrilabia_1682-1to3_SGTN	Single	2020	35.5	-85.5	3(2)		
Platanthera_integrilabia_1683-1_SGTN	Single	2020	35.5	-85.5	1(1)		
Platanthera_integrilabia_1684-1_SGTN	Single	2020	35.5	-85.5	1(1)	1(1)	
Platanthera_integrilabia_1755-1_MLAL	Single	2021	33.7	-85.8	1(1)		

Species_Sample#_LocationCode	Single Species or Mixed	Year	latitude	longitude	Cultures	SangerRoot	MetaRoot
Platanthera_integrilabia_1756_MLAL	Single	2021	33.7	-85.8			1(1)
Platanthera_integrilabia_1758-01_MLAL	Single	2021	33.7	-85.8	1(1)		
Platanthera_integrilabia_1777_CVKY	Single	2021	35.2	-87.0			1(1)
Platanthera_integrilabia_1778_CVKY	Single	2021	35.2	-87.0			1(1)
Platanthera_integrilabia_1779_CVKY	Single	2021	35.2	-87.0			1(1)
Platanthera_integrilabia_1780_CVKY	Single	2021	35.2	-87.0			1(1)
Platanthera_integrilabia_1781_EFKY	Single	2021	35.2	-86.9			1(1)
Platanthera_integrilabia_1782_CVKY	Single	2021	35.2	-87.0			1(1)
Platanthera_integrilabia_235_UKKY	Single	2018	35.8	-85.4	1(1)		
Platanthera_integrilabia_238_UKKY	Single	2018	35.8	-85.4	1(1)		
Platanthera_integrilabia_UAMH7632_UKKY	Single	2018	35.8	-85.4	1(1)		
Platanthera_lacera_0142-1-A-B_LSMD	Single	1998	38.9	-76.6	3(1)		
Platanthera_lacera_0547-R-1_MSVA	Single	2003	38.1	-78.8	1(1)	1(2)	
Platanthera_lacera_0580-1_MYVA	Single	2003	38.4	-79.6	1(1)		
Platanthera_lacera_0794-1-A-B_SXMD	Single	2010	38.9	-76.5	3(1)		
Platanthera_lacera_0795-A_SXMD	Single	2010	38.9	-76.5	1(1)		
Platanthera_lacera_0934-1-2_HCMD	Single	2016	39.3	-77.0	2(1)		
Platanthera_lacera_0993-1_NCDE	Single	2016	39.8	-75.6	1(1)		
Platanthera_lacera_1033_BFVA	Single	2016	37.4	-79.5			1(1)
Platanthera_lacera_1118-R-2-3_OZWI	Single	2017	43.4	-88.0	2(1)	1(2)	
Platanthera_lacera_1204-1-4_NCDE	Single	2017	39.8	-75.6	2(1)		
Platanthera_lacera_1295_VBPA	Single	2018	41.4	-70.7			1(2)
Platanthera_lacera_1549_DCMA	Single	2020	41.4	-70.7			1(1)
Platanthera_lacera_1550_DCMA	Single	2020	38.9	-76.5			1(1)
Platanthera_lacera_1570_BTMD	Single	2020	39.1	-77.0			1(1)
Platanthera_lacera_1571_MAMD	Single	2020	43.5	-71.4			1(1)
Platanthera_lacera_1572_GFNH	Single	2020	43.8	-71.3			1(1)
Platanthera_lacera_1578_TWNH	Single	2020	43.5	-71.4			1(1)
Platanthera_lacera_1583_GRNH	Single	2020	38.3	-80.2			1(1)
Platanthera_lacera_1589_CGWV	Single	2020	38.3	-80.2			1(1)
Platanthera_lacera_1592_CGWV	Single	2020	38.2	-80.4			1(1)
Platanthera_lacera_1593-R-3_CGWV	Single	2020	38.2	-80.4	1(1)	1(1)	
Platanthera_lacera_1808_TFPA	Single	2022	41.6	-77.3			1(1)
Platanthera_lacera_1970_SVMI	Single	2023	45.9	-88.0			1(1)
Platanthera_leucophaea_100_Iowa2016_Thixton	Single	2016	42.2	-90.5		1(1)	
Platanthera_leucophaea_101_Iowa2016_Thixton	Single	2016	42.2	-90.5		1(1)	
Platanthera_leucophaea_103_Iowa2016_Thixton	Single	2016	42.2	-90.5		1(1)	
Platanthera_leucophaea_104_Iowa2016_Thixton	Single	2016	42.2	-90.5		1(1)	
Platanthera_leucophaea_105_Iowa2016_Thixton	Single	2016	42.2	-90.5		1(1)	
Platanthera_leucophaea_1120-1-2_OZWI	Single	2017	43.4	-88.0	2(1)	1(2)	
Platanthera_leucophaea_114_Iowa2016_Thixton	Single	2016	42.2	-90.5		1(1)	
Platanthera_leucophaea_116Thixton	Single	2016	42.2	-90.5		1(1)	
Platanthera_leucophaea_196_HelmRoad2017Thixton	Single	2016	42.1	-88.2		1(1)	
Platanthera_leucophaea_198Helmroad_Thixton	Single	2016	42.1	-88.2		1(1)	
Platanthera_leucophaea_370_Michigan_Thixton	Single	2016	43.5	-83.5		1(1)	
Platanthera_leucophaea_70HelmRd_Thixton_XXXX	Single	2016	42.1	-88.2		1(1)	
Platanthera_leucophaea_HTB_114HT_Thixton	Single	2016	42.2	-90.5		1(1)	
Platanthera_leucophaea_JCIA	Single	2016	42.2	-90.5		1(1)	
Platanthera_leucophaea_L1ZET_370_fromMichigan_Thix	Single	2016	43.5	-83.5		1(1)	
Platanthera_leucophaea_N1L312MunsoncemeteryII_Thix	Single	2016	41.3	-90.3		1(1)	
Platanthera_leucophaea_NGIL	Single	2016	41.7	-89.3		1(1)	
Platanthera_leucophaea_Thixton_417_XXXX	Single	2016	42.3	-88.4	1(1)		
Platanthera_leucophaea_Thixton_418_XXXX	Single	2016	42.3	-88.4	1(1)		
Platanthera_limosa_1977_MLAZ	Single	2023	32.4	-110.8			1(1)
Platanthera_michaelii_1252-1-2_MCCA	Single	2018	38.1	-122.8	2(1)		
Platanthera_obtusata_0855-1_JRAK	Single	2013	59.6	-151.6	1(1)		
Platanthera_obtusata_0856_JRAK	Single	2013	59.6	-151.6			1(1)
Platanthera_obtusata_1019-1_BEMN	Single	2016	48.3	-94.7	1(1)		
Platanthera_obtusata_1478-1_RSWI	Single	2019	45.1	-87.1	1(1)		
Platanthera_obtusata_1479-1_WIWI	Single	2019	45.1	-87.1	1(1)		
Platanthera_obtusata_1480-1_WIWI	Single	2019	45.1	-87.1	1(1)		
Platanthera_obtusata_1604_TCMI	Single	2020	48.6	-95.2			1(1)
Platanthera_obtusata_1605_TCMI	Single	2020	48.6	-95.2			1(1)
Platanthera_obtusata_1946_RSWI	Single		45.1	-87.1			1(1)
Platanthera_obtusata_2067_1_NSMN	Single		48.6	-95.2	1(1)		
Platanthera_obtusata_2068_1_NSMN	Single		48.6	-95.2	1(1)		
Platanthera_obtusata_2245_1_OFMN	Single		47.6	-93.9	1(1)		
Platanthera_obtusata_1020-1_BEMN	Single	2016	48.3	-94.7	1(1)		
Platanthera_orbiculata_0552_PHWV	Single	2003	38.3	-79.9			1(1)

Species_Sample#_LocationCode	Single Species or Mixed	Year	latitude	longitude	Cultures	SangerRoot	MetaRoot
Platanthera_orbiculata_0755-A-B_LFVA	Single	2009	38.6	-79.9	2(1)		
Platanthera_orbiculata_0756-B-D-E_LFVA	Single	2009	38.6	-79.9	3(1)		
Platanthera_orbiculata_0761-A_LFVA	Single	2009	38.6	-79.9	1(1)		
Platanthera_orbiculata_0819-1-B-C_EPWV	Single	2010	38.4	-79.7	3(1)		
Platanthera_orbiculata_1061_SPWV	Single	2016	38.3	-79.9			1(1)
Platanthera_orbiculata_1062_SPWV	Single	2016	38.3	-79.9			1(2)
Platanthera_orbiculata_1063_SPWV	Single	2016	38.3	-79.9			1(2)
Platanthera_orbiculata_1108R-1_BGWV	Single	2017	37.9	-80.3	1(1)	1(2)	
Platanthera_orbiculata_1109-1_SPWV	Single	2017	38.3	-79.9	1(1)		
Platanthera_orbiculata_1211_GOMI	Single	2017	46.3	-89.5			1(1)
Platanthera_orbiculata_1720-01_TFPA	Single	2021	41.6	-77.5	1(1)		
Platanthera_orbiculata_1804_TFPA	Single	2022	41.6	-77.5			1(1)
Platanthera_orbiculata_1817_TFPA	Single	2022	41.6	-77.5			1(1)
Platanthera_orbiculata_1828_TFPA	Single	2022	41.6	-77.5			1(1)
Platanthera_orbiculata_1883_TFPA	Single	2022	41.6	-77.5			1(1)
Platanthera_orbiculata_1884_TFPA	Single	2022	41.6	-77.5			1(1)
Platanthera_orbiculata_548R-1_GBWV	Single	2003	38.3	-79.9	1(1)	1(1)	
Platanthera_orbiculata_PtO2or_TCPA	Single	2022	41.6	-77.5			1(2)
Platanthera_orbiculata_PtO4or_TCPA	Single	2022	41.6	-77.5			1(2)
Platanthera_orbiculata_PtO5_or_TCPA	Single	2022	41.6	-77.5			1(2)
Platanthera_orbiculata_PtO6or_TCPA	Single	2022	41.6	-77.5			1(2)
Platanthera_peramoena_0581-R-1_STVA	Single	2003	37.8	-77.6	1(1)	1(1)	
Platanthera_peramoena_0582-R-2_GOVA	Single	2003	38.0	-79.5	1(1)	1(1)	
Platanthera_peramoena_0588-R-1_STVA	Single	2003	38.0	-79.5	1(1)	1(1)	
Platanthera_peramoena_0589-R-1_HLVA	Single	2003	37.8	-77.6	1(1)	1(1)	
Platanthera_peramoena_0989_1_NCDE	Single	2016	39.5	-75.7	1(1)		
Platanthera_peramoena_1035_BEVA	Single	2016	38.3	-79.7			1(2)
Platanthera_peramoena_1036_BEVA	Single	2016	37.4	-79.5			1(1)
Platanthera_peramoena_1037_BFVA	Single	2016	37.4	-79.5			1(1)
Platanthera_peramoena_1292_MPMD	Single	2018	39.2	-76.8			1(1)
Platanthera_peramoena_1435_MPMD	Single	2019	39.2	-76.8			1(1)
Platanthera_peramoena_1439_MPMD	Single	2019	39.2	-76.8			1(2)
Platanthera_peramoena_1771_FCVA	Single	2021	39.0	-77.3			1(1)
Platanthera_peramoena_1773_FCVA	Single	2021	39.0	-77.3			1(1)
Platanthera_peramoena_1774_FCVA	Single	2021	39.0	-77.3			1(1)
Platanthera_peramoena_424_XXXX	Single		42.9	-91.9	1(1)		
Platanthera_peramoena_425_XXXX	Single		42.9	-91.9	1(1)		
Platanthera_praeclara_2145-1_BMMN	Single		49.2	-96.7	1(1)		
Platanthera_psycodes_475R-1-2_HLMA	Single	2002	42.6	-72.7	2(1)	1(2)	
Platanthera_psycodes_0507R-2_BSNY	Single	2002	42.4	-76.4	1(1)	1(2)	
Platanthera_psycodes_0583R-1_DMWV	Single	2003	38.3	-80.0	1(1)	1(1)	
Platanthera_psycodes_0595R-1-2_LIVA	Single	2003	38.9	-78.1	2(1)	1(1)	
Platanthera_psycodes_0751-A_THVA	Single	2009	38.4	-79.8	1(1)		
Platanthera_psycodes_0752-A-B-D_THVA	Single	2009	38.4	-79.8	3(1)		
Platanthera_psycodes_0815-A_THWV	Single	2010	38.4	-82.3	1(1)		
Platanthera_psycodes_816-B-C-E_THWV	Single	2010	38.4	-82.3	3(1)		
Platanthera_psycodes_0874-1-2_RENC	Single	2014	35.8	-82.3	2(1)		
Platanthera_psycodes_0876-1-2-3_RENC	Single	2014	35.8	-82.3	3(1)		
Platanthera_psycodes_1014_WCMA	Single	2016	42.4	-71.9			1(1)
Platanthera_psycodes_1015_WCMA	Single	2016	42.4	-71.9			1(1)
Platanthera_psycodes_1146_OZWI	Single	2017	43.7	-88.1			1(2)
Platanthera_psycodes_1341-2_RNWI	Single	2018	43.4	-88.0	1(1)		
Platanthera_psycodes_1346-1-2-3_ECVT	Mixed(grandiflora)	2018	44.3	-72.8	3(1)		
Platanthera_psycodes_1554_RCGA	Single	2020	35.1	-83.6			1(1)
Platanthera_psycodes_1558_WGGA	Single	2020	35.1	-83.6			1(1)
Platanthera_psycodes_1559_WGGA	Single	2020	35.1	-83.6			1(2)
Platanthera_psycodes_1560_WGGA	Single	2020	34.8	-84.0			1(1)
Platanthera_psycodes_1573_TBNH	Single	2020	43.7	-71.3			1(1)
Platanthera_psycodes_1574_TBNH	Single	2020	43.7	-71.3			1(1)
Platanthera_psycodes_1575_TBNH	Single	2020	43.7	-71.3			1(1)
Platanthera_psycodes_1577_CHNH	Single	2020	43.9	-71.2			1(1)
Platanthera_psycodes_1712_LCIL	Single	2021	42.2	-87.8			1(1)
Platanthera_psycodes_1713_LCIL	Single	2021	42.2	-87.8			1(1)
Platanthera_psycodes_1739-02_GPMN	Single	2021	47.3	-93.5	1(1)		
Platanthera_psycodes_1740-01_GPMN	Single	2021	47.3	-93.5	1(1)		
Platanthera_psycodes_1741-01_GPMN	Single	2021	47.3	-93.5	1(1)		
Platanthera_psycodes_1748R-1-4_CRMN	Single	2021	47.7	-90.5	2(1)		
Platanthera_psycodes_1749_CRMN	Single	2021	47.7	-90.5			1(2)
Platanthera_psycodes_1750-01_CRMN	Single	2021	47.7	-90.5	2(2)		

Species_Sample#_LocationCode	Single Species or Mixed	Year	latitude	longitude	Cultures	SangerRoot	MetaRoot
Platanthera_psycodes_1954_TRMI	Single	2023	46.5	-85.3			1(1)
Platanthera_psycodes_1955_CPMI	Single	2023	45.6	-84.4			1(2)
Platanthera_psycodes_1956_CPMI	Single	2023	45.6	-84.4			1(2)
Platanthera_psycodes_2077_HMMI	Single	2024	46.9	-87.8			1(2)
Platanthera_psycodes_2089_HMMI	Single	2024	46.9	-87.8			1(2)
Platanthera_psycodes_503_BSNY	Single	2002	42.4	-76.4			1(2)
Platanthera_shriveri_0753-A_LFVA	Single	2009	38.7	-79.7	1(1)		
Platanthera_shriveri_0813_AMWV	Mixed(grandiflora)	2010	38.3	-80.2		1(1)	
Platanthera_shriveri_0814_AMWV	Mixed(grandiflora)	2010	38.3	-80.2		1(2)	
Platanthera_shriveri_1511_FCMD	Single	2019	39.6	-77.5			1(1)
Platanthera_shriveri_1512_FCMD	Single	2019	39.6	-77.5			1(1)
Platanthera_shriveri_1587-1-2_WRWV	Mixed(grandiflora)	2020	38.2	-80.2	2(1)		
Platanthera_shriveri_1588_1_WRWV	Mixed(grandiflora)	2020	38.2	-80.2	1(1)		
Platanthera_sparsiflora_1477-1_HMCA	Single	2019	40.9	-123.7	1(1)		
Platanthera_x_bicolor_0731-C-E_VBPA	Mixed(blephariglottis, ciliaris)	2009	41.0	-76.0	3(2)		
Platanthera_x_bicolor_0736-1-A_VBPA	Mixed(blephariglottis, ciliaris)	2009	41.0	-76.0	2(2)		
Platanthera_x_bicolor_0738-A_VBPA	Mixed(blephariglottis, ciliaris)	2009	41.0	-76.0	1(1)		
Platanthera_x_bicolor_1242-1-2_VBPA	Mixed(blephariglottis, ciliaris)	2018	41.0	-76.0	2(1)		
Platanthera_x_bicolor_1243_VBPA	Mixed(blephariglottis, ciliaris)	2018	41.0	-76.0			1(1)
Platanthera_x_bicolor_1244_VBPA	Mixed(blephariglottis, ciliaris)	2018	41.0	-76.0			1(1)
Platanthera_x_bicolor_1245-1-2_VBPA	Mixed(blephariglottis, ciliaris)	2018	41.0	-76.0	2(1)		
Platanthera_x_bicolor_1246-1_VBPA	Mixed(blephariglottis, ciliaris)	2018	41.0	-76.0	1(1)		
Platanthera_x_bicolor_1247-2(3)_VBPA	Mixed(blephariglottis, ciliaris)	2018	41.0	-76.0	1(1)		
Platanthera_x_bicolor_1290_VBPA	Mixed(blephariglottis, ciliaris)	2018	41.0	-76.0		1(2)	
Platanthera_x_bicolor_1296_VBPA	Mixed(blephariglottis, ciliaris)	2018	41.0	-76.0		1(1)	
Platanthera_x_bicolor_1297_VBPA	Mixed(blephariglottis, ciliaris)	2018	41.0	-76.0		1(1)	
Platanthera_x_bicolor_1372-1_VBPA	Mixed(blephariglottis, ciliaris)	2018	41.0	-76.0	1(1)		
Platanthera_x_bicolor_1424_VBPA	Mixed(blephariglottis, ciliaris)	2019	41.0	-76.0			1(2)
Platanthera_x_bicolor_1425-1-2-3_VBPA	Mixed(blephariglottis, ciliaris)	2019	41.0	-76.0	3(1)		
Platanthera_x_bicolor_1453_GSNC	Mixed(blephariglottis, ciliaris, cristata)	2019	34.1	-78.3			1(1)
Platanthera_x_bicolor_1457_GSNC	Mixed(blephariglottis, ciliaris, cristata)	2019	34.1	-78.3			1(2)
Platanthera_x_bicolor_1460_GSNC	Mixed(blephariglottis, ciliaris, cristata)	2019	34.1	-78.3			1(1)
Platanthera_x_bicolor_1731-01_VBPA	Mixed(blephariglottis, ciliaris)	2021	41.0	-76.0	1(1)		
Platanthera_x_bicolor_1732_VBPA	Mixed(blephariglottis, ciliaris)	2021	41.0	-76.0			1(1)
Platanthera_x_bicolor_1734_VBPA	Mixed(blephariglottis, ciliaris)	2021	41.0	-76.0			1(1)
Platanthera_x_bicolor_1735_VBPA	Mixed(blephariglottis, ciliaris)	2021	41.0	-76.0			1(1)
Platanthera_x_bicolor_1911-1_RBPA	Mixed(blephariglottis, ciliaris)	2023	41.0	-76.0	1(1)		
Platanthera_x_bicolor_1916-1_RBPA	Mixed(blephariglottis, ciliaris)	2023	41.0	-76.0	1(1)		
Platanthera_x_bicolor_1963_AGMI	Mixed(blephariglottis, ciliaris)	2023	42.6	-86.1			1(1)
Platanthera_x_bicolor_1964_AGMI	Mixed(blephariglottis, ciliaris)	2023	42.6	-86.1			1(2)
Platanthera_x_bicolor_1966_AGMI	Mixed(blephariglottis, ciliaris)	2023	42.6	-86.1			1(2)
Platanthera_x_canbyi_0772-A_NBMD	Mixed(blephariglottis, cristata)	2009	38.3	-75.5	1(2)		
Platanthera_x_canbyi_0773-A_NBMD	Mixed(blephariglottis, cristata)	2009	38.3	-75.5	1(1)		
Platanthera_x_canbyi_1301-2_NBMD	Mixed(blephariglottis, cristata)	2018	38.3	-75.5	1(1)		
Platanthera_x_canbyi_1410_NBMD	Mixed(blephariglottis, cristata)	2019	38.3	-75.5			1(1)
Platanthera_x_canbyi_1411_NBMD	Mixed(blephariglottis, cristata)	2019	38.3	-75.5			1(2)
Platanthera_x_canbyi_1418_PINJ	Mixed(blephariglottis, cristata)	2019	39.7	-74.5			1(1)
Platanthera_x_canbyi_1419_PINJ	Mixed(blephariglottis, cristata)	2019	39.7	-74.5			1(1)
Platanthera_x_canbyi_1746_PINJ	Mixed(blephariglottis, cristata)	2021	39.7	-74.5			1(1)
Platanthera_x_canbyi_0643R-1_NBMD	Mixed(blephariglottis, cristata)	2004	38.3	-75.5	1(1)		
Platanthera_x_canbyi_0646-1_NBMD	Mixed(blephariglottis, cristata)	2004	38.3	-75.5	1(1)		
Platanthera_ciliaris_x_integrilabia_1697-1_HMKY	Mixed(ciliaris, integrilabia)	2020	37.2	-84.5	1(1)		
Platanthera_x_keenanii_0554-R-1_GBWV	Mixed(grandiflora, lacera)	2003	37.8	-80.3	1(2)	1(1)	
Platanthera_x_keenanii_549_GBWV	Mixed(grandiflora, lacera)	2003	37.8	-80.3			1(2)
