# Supplementary material for: Mycorrhizal fungal associations of the fringed orchids (Platanthera) in the US, inter- and intra-species variation
Source: Biodivers Conserv. 2026 Jan 23;35(2):48. doi: 10.1007/s10531-025-03233-4 (PMC12827425; doi:10.1007/s10531-025-03233-4)
Supplement: Supplementary file 5 — Supplementary Material 5 [file 10531_2025_3233_MOESM5_ESM.pdf]

Table S2: Sequencing method that yielded reads of *Ceratobasidium* (Cer) or *Tulasnella* (Tul) for each *Platanthera* species. Note that all sequencing methods were not attempted on all samples. Previously published sequences were obtained from GenBank or from the study's author. Cultures were all sequenced using Sanger sequencing. "Culture and Root" refers to samples where both cultures and roots from a single sample were sequenced using multiple sequencing and identical OTUs were obtained. This was rarely attempted, because we prioritized samples without sequences, when conducting more sequencing. However, when identical sequences were obtained using multiple methods the reads were combined into a single read for further analysis.

| | **Sequencing Method** | | | | | | | | | |
|---|---|---|---|---|---|---|---|---|---|---|
| | Previously published | | Cultures | | Sanger | | Metabarcoding | | Culture and Root | |
| | *Cer.* | *Tul.* | *Cer.* | *Tul.* | *Cer.* | *Tul.* | *Cer.* | *Tul.* | *Cer.* | *Tul.* |
| *Platanthera holochila* | | 3 | | | | | | | | |
| *Platanthera aquilonis* | | | | | 1 | 1 | 1 | | | |
| *Platanthera blephariglottis* | | | 6 | 19 | 3 | 21 | 17 | 15 | | 4 |
| *Platanthera chapmanii* | 5 | 139 | | 5 | | 9 | 5 | 141 | | |
| *Platanthera ciliaris* | | | 7 | 43 | 4 | 55 | 15 | 20 | | 7 |
| *Platanthera clavellata* | | | 12 | 31 | 7 | 46 | 19 | 22 | 2 | 5 |
| *Platanthera convallariafolia* | | | | | | | | 2 | | |
| *Platanthera cooperi* | | 11 | | | | | | 11 | | |
| *Platanthera cristata* | | | 2 | 11 | 5 | 14 | 15 | 7 | | 5 |
| *Platanthera dilatata* | | | | 6 | | 9 | | 1 | | |
| *Platanthera flava* | | | 5 | 14 | 4 | 21 | 10 | 2 | 1 | 3 |
| *Platanthera grandiflora* | | | 12 | 17 | 10 | 20 | 14 | 5 | 2 | 3 |
| *Platanthera hookeri* | | | 2 | 1 | 2 | 4 | | | | |
| *Platanthera huronensis* | | | 3 | 1 | 3 | 2 | | | | |
| *Platanthera hyperborea* | | | | 2 | | 3 | | | | |
| *Platanthera integra* | | | 1 | 4 | 1 | 4 | | | | 2 |
| *Platanthera integrilabia* | | | | 9 | | 14 | 1 | 1 | | 1 |
| *Platanthera lacera* | | 1 | 15 | 4 | 10 | 5 | 15 | 1 | 5 | |
| *Platanthera leucophaea* | 21 | 2 | | 2 | | 3 | 23 | 3 | | |
| *Platanthera limosa* | | | | | 1 | 1 | 1 | | | |
| *Platanthera michaelii* | | | | 1 | | 1 | | | | |
| *Platanthera obtusata* | | | 3 | 6 | 2 | 7 | 3 | 1 | | |
| *Platanthera orbiculata* | | | 1 | 15 | 1 | 20 | | 4 | | 4 |
| *Platanthera peramoena* | | | 1 | 7 | 1 | 14 | 4 | 1 | | 4 |
| *Platanthera praeclara* | 127 | 27 | | | | | 127 | 27 | | |
| *Platanthera psycodes* | | | 10 | 19 | 9 | 25 | 18 | 8 | 1 | 5 |
| *Platanthera shriveri* | | | 2 | 5 | 2 | 6 | 2 | | | |
| *Platanthera sparsiflora* | | | | 1 | | 1 | | | | |
| *Platanthera x bicolor* | | | 1 | 14 | 1 | 20 | 9 | 5 | | 2 |
| *Platanthera x canbyii* | | | 1 | 7 | 1 | 9 | 4 | | | 2 |
| *Platanthera x integrilabia* | | | | 1 | | 1 | | | | |
| *Platanthera x keenanii* | | | | 1 | | 2 | | | | 1 |
| *Platanthera yadonii* | 31 | 48 | 1 | | 1 | | | | | |
